# Implementing the Quantum Fourier Transform on a molecular qudit with full refocusing and state tomography

**DOI:** 10.1038/s41467-026-72390-z

**Published:** 2026-05-07

**Authors:** Marcos Rubín-Osanz, Laura Bersani, Simone Chicco, Giuseppe Allodi, Roberto De Renzi, Athanasios Mavromagoulos, Michael D. Roy, Stergios Piligkos, Elena Garlatti, Stefano Carretta

**Affiliations:** 1 Dipartimento di Scienze Matematiche, Fisiche e Informatiche, Università di Parma, I-43124 Parma, Italy; 2 Department of Chemistry, University of Copenhagen, DK-2100 Copenhagen, Denmark; 3 UdR Parma, INSTM, I-43124 Parma, Italy; 4 INFN, Sezione di Milano-Bicocca, gruppo collegato di Parma, I-43124 Parma, Italy

**Keywords:** Magnetic properties and materials, Qubits, Solid-state NMR, Inorganic chemistry

## Abstract

Molecular spin qudits based on lanthanide complexes offer a promising platform for quantum technologies, combining chemical tunability with multi-level encoding. However, experimental demonstrations of their envisaged capabilities remain scarce, posing the difficulty of achieving precise control over coherences between qudit states in long pulse sequences. Here, we implement in a ^173^Yb(trensal) qudit the Quantum Fourier Transform (QFT), a core component of numerous quantum algorithms, storing quantum information in the phases of coherences. QFT provides an ideal benchmark for coherence manipulation and a challenge for molecular spin qudits. We address this challenge by embedding a full-refocusing protocol for spin qudits in our algorithm, mitigating inhomogeneous broadening and enabling a high-fidelity recovery of the state. Complete state tomography demonstrates the performance of the algorithm, while simulations provide insight into the physical mechanisms behind inhomogeneous broadening. This work shows the feasibility of quantum logic on molecular spin qudits and highlights their potential.

## Abstract (web summary)

Researchers implement quantum Fourier transform on a 173Yb (trensal) molecular spin qudit, adding embedded refocusing to supress ensemble dephasing from inhomogeneous broadening. By keeping fragile signals intact, the team shows this approach can work in practice.

## Introduction

The experimental realization of quantum algorithms provides a crucial benchmark for assessing the potential of emerging quantum platforms. Most demonstrations have focused on qubit-based architectures such as superconducting circuits^1–3^, trapped ions^4–6^, and semiconductor quantum dots^7–9^, prompting the second quantum revolution. Multi-level quantum systems exploited as qudits—quantum digits with dimension *d* > 2—offer an alternative that has recently gained increasing attention^10–13^ due to its ability to encode higher-dimensional Hilbert spaces in a single physical system, reducing circuit depth and enabling more efficient quantum error-correction protocols. Indeed, incorporating quantum error correction and fault-tolerant logic into qudit-based architectures is particularly promising to unlock the full potential of quantum computing in the Noisy Intermediate-Scale Quantum (NISQ) era, where coherence times and gate fidelities remain limited^14–21^.

Among the available physical platforms, molecular spin qudits (MSQs) represent an appealing route to quantum technologies thanks to their chemical tunability, allowing them to have both a large number of accessible levels and long coherence times^22–25^. In particular, hyperfine-split levels in single-ion systems provide a natural multi-level structure that can be coherently controlled using advanced radio-frequency (RF) pulse sequences^26–28^. The hyperfine manifold of even simple single-ion systems like ^173^Yb(trensal) provides, in fact, up to *d* = 12 electro-nuclear spin states, with sizeable hyperfine gaps, enabling thermal initialization below 15 mK. The design of MSQs with a nuclear spin—naturally encoding a qudit—coupled to the effective molecular electronic spin also provides a pathway for scalability through the coupling of the latter to the magnetic^29^ and electric^30,31^ fields of superconducting resonators in circuit quantum electrodynamics architectures^32,33^.

The remarkable potential of MSQs has been thoroughly investigated theoretically^25^, but solid experimental validations of some fundamental steps towards a molecule-based quantum computer are still very few^28,34,35^. In particular, in a recent work, we provided significant experimental evidence of their capabilities by realizing the proof-of-concept implementation of a quantum simulator with an ensemble of MSQs^28^. The accurate quantum simulation of the time evolutions of different target Hamiltonians had the advantage of requiring only the measurement of output populations of the qudit, whereas a demonstration of a full algorithmic control demands the verification of both populations and coherences (off-diagonal elements in the density matrix) of the output state of the qudit, the latter being more prone to errors due to various sources of dephasing.

In this work, we experimentally implemented the Quantum Fourier Transform (QFT) on the hyperfine manifold of the molecular spin qudit ^173^Yb(trensal). The QFT is a fundamental primitive in quantum algorithms such as phase estimation and Shor’s algorithm^36^, and its high-fidelity execution requires precise control over coherences across the entire density matrix, where quantum information is stored. Our molecules are magnetically diluted in a single crystal, ensuring coherence times *T*_2_ > 0.1 ms, while typical driving pulse durations are of a few hundred ns for the relevant transitions. However, working with a molecular ensemble means that inhomogeneous broadening becomes a significant source of dephasing. In fact, typical characteristic times ($${T}_{2}^{*} < $$1 μs) for this effect are comparable to the duration of a short 3-pulse sequence, hindering the direct implementation of the QFT. We addressed this problem with a full-refocusing scheme tailored for MSQs, embedded in the pulse sequence implementing the QFT, that allows the recovery of coherences even after complex quantum operations consisting of more than 20 pulses. We demonstrated the performance of our refocused QFT algorithm via complete quantum state tomography, achieving high fidelity of the output qudit state. Additionally, our measurements and simulations provide insight into the microscopic mechanisms responsible for inhomogeneous broadening in our MSQs.

This work represents a key step towards practical implementations of quantum logic operations on MSQs, demonstrating algorithmic control and coherence preservation with high fidelity in complex pulse sequences. The sequences designed here, including the refocusing scheme, can be generalized to qudits with *d* > 3 and combined with optimization techniques (e.g., pulse shaping to minimize leakage outside of the desired quantum subspace)^37,38^ to enhance the performance of our hardware. Thus, our results place molecular spin qudit platforms as promising candidates for qudit-based quantum technologies.

## Results

### Characterization and calibration of the qudit

**Fig. 1:**
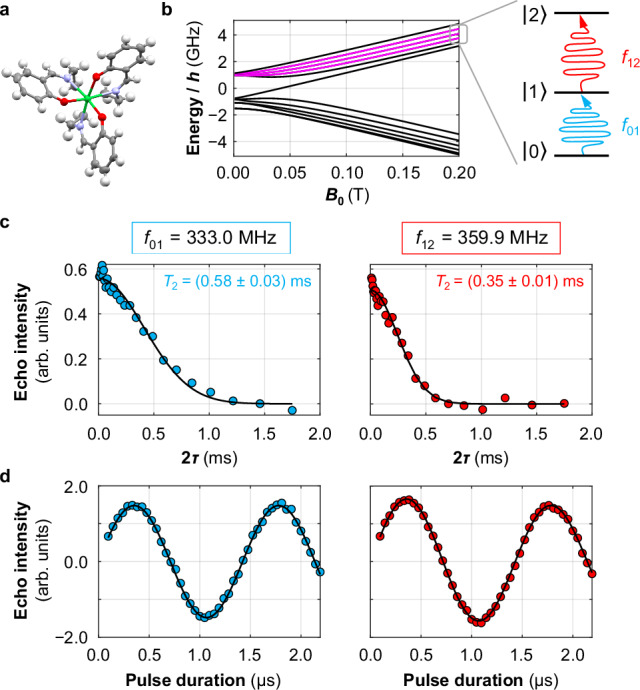
Characterization of the qudit. **a** Molecular structure of ^173^Yb(trensal). **b** Energy level diagram of ^173^Yb(trensal) in a static magnetic field **B**_0_ perpendicular to the molecular C_3_ axis. The states of the subspace targeted in this work, highlighted in pink, are labeled $\left|0\right\rangle$, $\left|1\right\rangle$ and $\left|2\right\rangle$ from lowest to highest energy, with transition frequencies *f*_01_ and *f*_12_. **c** Phase memory time *T*_2_ of both transitions, measured at *B*_0_ = 0.2 T and *T* = 1.4 K (scatters) and fit to a Gaussian-shaped decay (solid line). **d** Coherent Rabi manipulation of both transitions under these same experimental conditions. The black solid line is the fit of the Rabi oscillations, which gives a *π*-pulse duration of 750 ns for the spectrometer. Error bars on all data are of the order of scatter dimension; they were obtained by applying to the noise the same analysis we applied to the data (see “Methods”) and taking the root mean square.

Our platform is an isotopically enriched single crystal of ^173^Yb(trensal), diluted at 0.05% in its diamagnetic isostructural analog Lu(trensal) to reduce intermolecular magnetic interactions, see “Methods”. At this dilution, each ^173^Yb(trensal) molecule^39,40^ (Fig. 1a) behaves as an isolated qudit^26^ consisting of an effective electronic spin *S* = 1/2 coupled to the nuclear spin *I* = 5/2 of the ^173^Yb isotope, for a total of 12 energy levels (Fig. 1b) described by the effective Hamiltonian (in frequency units)

1 $${H}_{0}= \,{A}_{\parallel }{S}_{z}{I}_{z}+{A}_{\perp }({S}_{x}{I}_{x}+{S}_{y}{I}_{y})+p{I}_{z}^{2}\,+\left(\frac{{\mu }_{{{{\rm{B}}}}}}{h}\right){{{\bf{S}}}}\cdot {{{\bf{g}}}}\cdot {{{{\bf{B}}}}}_{0}-\left(\frac{{\mu }_{{{{\rm{N}}}}}}{h}\right){g}_{I}{{{\bf{I}}}}\cdot {{{{\bf{B}}}}}_{0}\,.$$

In Equation (1), **S** and **I** represent the electronic and nuclear spin operators, respectively (with components on the molecular axes $\widehat{x},\widehat{y},\widehat{z}$), and the first two terms are their strong hyperfine interaction, axial on the *C*_3_ symmetry axis ($\widehat{z}$) of the molecule, with hyperfine constants *A*_∥_ = − 883 MHz and *A*_⊥_ = − 628 MHz. The third term describes the nuclear quadrupolar coupling (*p* = − 66 MHz), while the last two are the electronic and nuclear Zeeman couplings to an external static magnetic field **B**_0_, with the electronic coupling being axial on the *C*_3_ axis (*g*_*x*_ = *g*_*y*_ = 2.9, *g*_*z*_ = 4.3) and the nuclear one isotropic (*g*_*I*_ = − 0.2592)^26,32^. These parameters generate an energy spectrum with distinct transition frequencies that allow both a selective, single transition manipulation and a multiple transition addressing in a broadband setup.

For the implementation of the QFT algorithm, the single crystal was inserted inside the coil of a custom multi-frequency probe and placed at the center of the bore of a 9 T superconducting magnet. The crystal was aligned with a custom non-magnetic sample holder to set the *C*_3_ axis of the ^173^Yb(trensal) molecules perpendicular to the magnetic field **B**_0_ inside the magnet bore (${{{{\bf{B}}}}}_{{{0}}}\parallel \widehat{x}$). We set *B*_0_ = 0.2 T, for which the electronic Zeeman is the dominant interaction in Eq.(1). This field splits the 12 states into two energy manifolds, with each state almost factorized in the electronic and nuclear spin subspaces, allowing a simple labeling in terms of the electronic and nuclear spin components along $\widehat{x}$: $\left|{m}_{S} , {m}_{I}\right\rangle$. We selected the 3-level subspace of the upper energy manifold highlighted in pink in Fig. 1b, which consisted of states $\left|0\right\rangle=\left|+\frac{1}{2},+\frac{1}{2}\right\rangle$, $\left|1\right\rangle=\left|+\frac{1}{2}, -\frac{1}{2}\right\rangle$, and $\left|2\right\rangle=\left|+\frac{1}{2}, -\frac{3}{2}\right\rangle$. Any operation limited to this subspace can also be understood as a controlled gate acting on the nuclear spin state only if *m*_*S*_ = + 1/2. This is possible in ^173^Yb(trensal) thanks to the distinct nuclear transition frequencies of the *m*_*S*_ = + 1/2 and *m*_*S*_ = − 1/2 manifolds. The two addressable transitions $\left|0\right\rangle \leftrightarrow \left|1\right\rangle$ and $\left|1\right\rangle \leftrightarrow \left|2\right\rangle$, with frequencies *f*_01_ = 333.0 MHz and *f*_12_ = 359.9 MHz, were the only ones within the frequency bandwidth (320 − 370 MHz) of the custom probe designed for the experiment, mitigating leakage to states outside this subspace. A comparison between the measured spectrum, the probe bandwidth and the frequencies of neighbor transitions is reported in the Supplementary Note 1. The system was cooled to *T* = 1.4 K, where we measured the relaxation (*T*_1_)—see Supplementary Note 1—and coherence (*T*_2_) times of both transitions. A remarkable feature of ^173^Yb(trensal) at this dilution and temperature is its long coherence times (*T*_2_ > 0.1 ms), as shown in Fig. 1c. Thus, the effect of pure dephasing in our results is small, as *T*_2_ is much longer than sequence duration. The Gaussian shape of the decay of coherences, typical of decoherence processes induced mainly by the degrees of freedom of the nuclear spin bath, is expected if nuclear dipolar interactions are the dominant dephasing mechanism^41^.

### Coherent control and qutrit state tomography

**Fig. 2:**
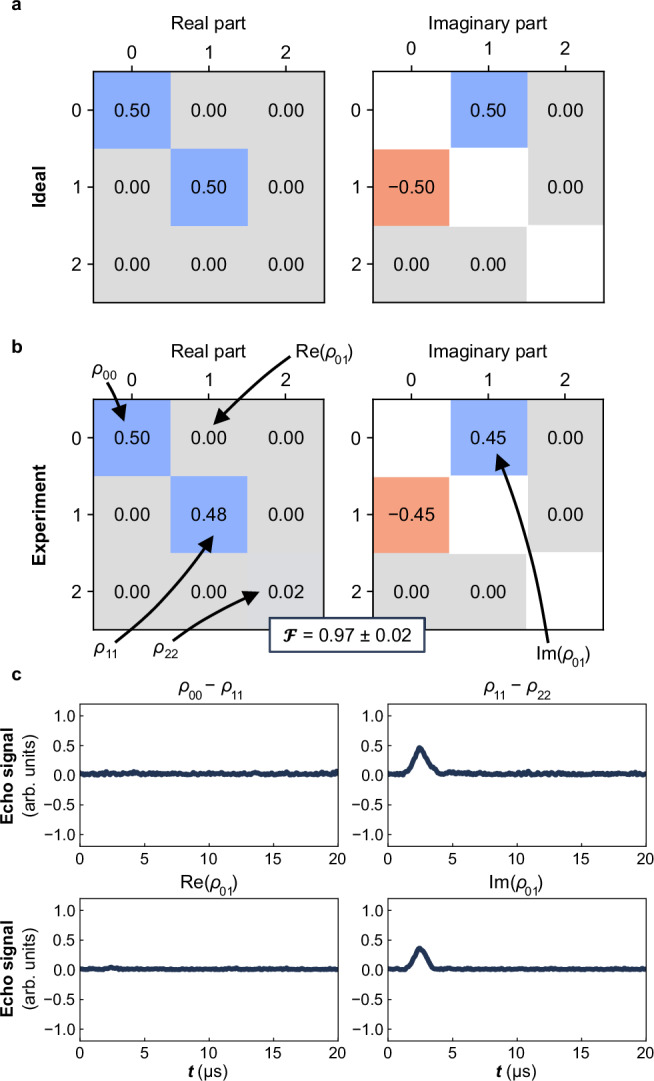
Coherent manipulation and tomography of the qutrit state. **a** Density matrix ${\rho }_{{{{\rm{ideal}}}}}=\left|\psi \right\rangle \left\langle \psi \right|$ for the ideal superposition state $\left|\psi \right\rangle=\frac{1}{\sqrt{2}}(\left|0\right\rangle -i\left|1\right\rangle )$. **b** Density matrix ${\rho }_{\exp }$ for the final state after the experimental manipulation of the initial state of the qutrit, $\left|0\right\rangle$, with a *θ* = *π*/2 pulse with phase *ϕ* = 0 and carrier frequency *f*_01_. This pulse ideally generates the superposition state of panel a. The elements of ${\rho }_{\exp }$ were measured by performing a complete tomography experiment of the final state. **c** Measured echo traces yielding the tomography of panel b. We estimated the error in ${{{\mathcal{F}}}}$ by applying the same noise analysis of Fig. 1 to these traces.

Coherent control was achieved via RF pulses, sent with an Arbitrary Waveform Generator (AWG) by Active Technologies and the home-made broadband spectrometer HyReSpect^42^, as demonstrated by the Rabi curves in Fig. 1d. Each pulse ideally produces a planar rotation in one of the two target transitions, defined as

2 $${P}_{\mu \nu }\left(\theta,\phi \right)=\cos \left(\frac{\theta }{2}\right)\left(\left|\mu \right\rangle \left\langle \mu \right|+\left|\nu \right\rangle \left\langle \nu \right|\right) -i\sin \left(\frac{\theta }{2}\right)\left({e}^{i\phi }\left|\nu \right\rangle \left\langle \mu \right|+{e}^{-i\phi }\left|\mu \right\rangle \left\langle \nu \right|\right)\,,$$

implementing a single qutrit gate involving states $\left|\mu \right\rangle$ and $\left|\nu \right\rangle$. The tilt angle *θ* of the rotation is controlled by the RF power, duration and shape of the pulse; the phase *ϕ* is that of the pulse carrier signal of frequency *f*_*μν*_. As an example, the ideal effect of a single pulse with $\theta=\frac{\pi }{2}$, *ϕ* = 0 and carrier frequency *f*_01_ on state $\left|0\right\rangle$, which generates the superposition state $\left|\psi \right\rangle= {P}_{01}\left(\pi /2, 0\right)\left|0\right\rangle= \frac{1}{\sqrt{2}}\left(\left|0\right\rangle -i\left|1\right\rangle \right)$, is shown in Fig. 2a as the density matrix ${\rho }_{{{{\rm{ideal}}}}}= \left|\psi \right\rangle \left\langle \psi \right|$. Our experimental implementation of the same gate yielded the density matrix ${\rho }_{\exp }$ in Fig. 2b, obtained by performing a full tomography experiment on the final superposition state. This demonstrates our coherent control of the qutrit state beyond Rabi experiments, with a gate fidelity^43^${{{\mathcal{F}}}}={{{\rm{Tr}}}}\left(\sqrt{\sqrt{{\rho }_{{{{\rm{ideal}}}}}}{\rho }_{\exp }\sqrt{{\rho }_{{{{\rm{ideal}}}}}}}\right)=0.97\pm 0.02$. Our gate and sequence fidelity definitions always include the fidelity of the tomography procedure, which, in fact, is one of the limitations of our current setup. Figure 2c shows the echo traces used to reconstruct ${\rho }_{\exp }$. See “Methods” for more details on the tomography experiments.

As the temperature of the system was still too high for a thermal initialization to a pure state, we generated a pseudo-pure state before every state manipulation experiment^28^. To do so, we sent a $\frac{\pi }{2}$ pulse with *f*_12_, equipopulating states $\left|1\right\rangle$ and $\left|2\right\rangle$, and let the coherence between the two states decay for a time *t* ≫ *T*_2_. The resulting density matrix in the $\left\{\left|0\right\rangle,\left|1\right\rangle,\left|2\right\rangle \right\}$ subspace can then be decomposed into a term proportional to the identity matrix, unaffected by further manipulation pulses, and a term proportional to a pure state, in the form: ${\rho }_{0-2}^{{\prime} }=\,{\alpha }_{0}\left|0\right\rangle \left\langle 0\right|\,+\,\frac{{p}_{1}+{p}_{2}}{2}\,{\mathbb{1}}$, with ${\alpha }_0={p}_0 {-}({p}_1+{p}_2)/2 \, {{\rm{and}}}\, {p}_\eta (\eta= 0, 1, 2)$ the initial Boltzmann population of the energy states. Thus, measurements on a pseudo-pure state are effectively equivalent to those on a pure state up to a normalization factor (see “Methods”).

### Implementation of the QFT

The QFT operation on a qudit with *d* states is described by the unitary transformation

3 $${U}_{d}=\frac{1}{\sqrt{d}}{\sum }_{\mu,\nu=0}^{d-1}{e}^{i2\pi \frac{\mu \nu }{d}}\left|\mu \right\rangle \left\langle \nu \right|.$$

**Fig. 3:**
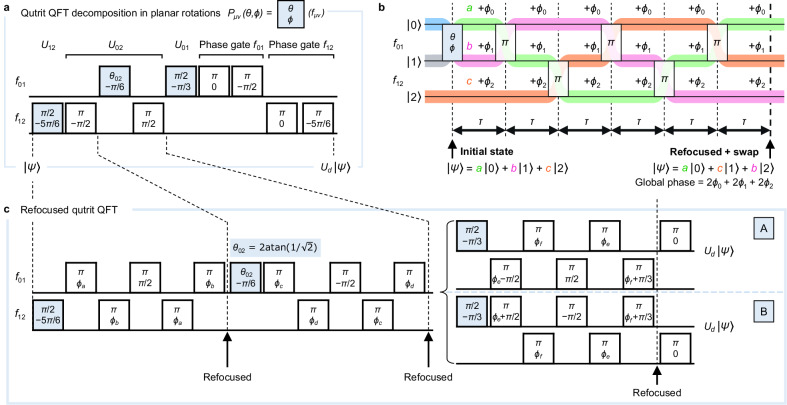
Pulse sequence for the QFT. **a** Decomposition of the 3-level unitary transformation *U*_*d*_ performing the qutrit QFT into 9 planar rotations. **b** Detail of a qutrit refocusing block. After a pulse (*θ*, *ϕ*) of the QFT sequence, a state $\left|\psi \right\rangle=a\left|0\right\rangle+b\left|1\right\rangle+c\left|2\right\rangle$ is encoded into the three-level subspace with amplitudes *a* (green), *b* (pink), and *c* (orange). Due to inhomogeneous broadening, the free evolution of each spin in the ensemble is slightly different, and each amplitude of each spin collects a different phase during the time *τ* between pulses. This phase depends on the basis state ($\left|0\right\rangle$, $\left|1\right\rangle$, $\left|2\right\rangle$) in which the amplitude is encoded (*ϕ*_0_, *ϕ*_1_ and *ϕ*_2_, respectively). A *π* pulse swaps the amplitudes between the basis states. By applying a sequence of five *π* pulses, equally spaced in time, amplitudes are moved around all the basis states, thus collecting the same global phase 2*ϕ*_0_ + 2*ϕ*_1_ + 2*ϕ*_2_. This final refocused state includes a swap between two states. **c** Pulse sequence implementing *U*_*d*_ with embedded refocusing blocks (see panel (**b**)) for any pulse with *θ* ≠ *π*. Any such pulse must be sent to a refocused state after a refocusing block. Conversely, *π*-pulses can be integrated in them to reduce the number of pulses. Only relative phases between some of the *π*-pulses are set by the QFT operation, we can freely choose *ϕ*_*a*_, *ϕ*_*b*_, *ϕ*_*c*_, *ϕ*_*d*_, *ϕ*_*e*_ and *ϕ*_*f*_. All results here were obtained with *ϕ*_*a*_ = *ϕ*_*b*_ = *ϕ*_*c*_ = *ϕ*_*d*_ = *ϕ*_*e*_ = 0 and *ϕ*_*f*_ = − *π*/6. Detection was performed on a refocused state, before the last *π* pulse that only introduces a swap between basis states. Both sequences A and B implement the QFT, each one allowing for a simple detection of different density matrix elements (see “Methods”).

The implementation of *U*_*d*_ for a qutrit (*d* = 3), depicted in Fig. 3a, consists of a sequence of optimized RF pulses based on its decomposition into planar rotations ${P}_{\mu \nu }\left(\theta,\phi \right)$ in the two addressable transitions^44^. However, measuring the result of this sequence in an ensemble of MSQs is greatly affected by inhomogeneities in the sample: coherences are lost in a timescale ${T}_{2}^{*}\ll {T}_{2}$ during the computation. For ^173^Yb(trensal), in the dilution and temperature conditions of these experiments, both transitions had ${T}_{2}^{*} \sim 500$ ns, determined from the width of the measured echoes (see Supplementary Note 2). This is comparable to the timescale of our pulses, and therefore significantly shorter than the whole sequence (9 pulses), reducing the fidelity of the implementation. In this regard, the QFT is a very stringent test for experiments with a molecular ensemble, as it generates all-to-all coherences and encodes quantum information in their relative phases.

The effect of inhomogeneous broadening can be mitigated by implementing a refocusing scheme for qutrits^45^. An example of a sequence that refocuses the qutrit state is depicted in Fig. 3b: after applying an arbitrary pulse, which generates a state $\left|\psi \right\rangle=a\left|0\right\rangle+b\left|1\right\rangle+c\left|2\right\rangle$, a sequence of five *π*-pulses alternating between the two addressable transitions transforms any phases generated by the free evolution of each state of each spin into a global phase, and swaps two states. A similar strategy has been implemented on qutrits encoded in cold atoms and superconducting processors to refocus a previously generated superposition qutrit state^46–48^. However, our relatively short ${T}_{2}^{*}$ requires embedding the refocusing in the manipulation of the qutrit state. For this, we designed the 19-pulse sequences in Fig. 3c, which implement the QFT while refocusing the qutrit state every 6 pulses. Numerical simulation of the full pulse-sequence on a ensemble of ^173^Yb(trensal) molecules, each one described by the Hamiltonian of Eq.(1) plus a strain in the Hamiltonian parameters (sampled from a Gaussian distribution around their mean values), yielded an almost perfect recovery of the ideal QFT operation in the absence of other sources of errors (see Supplementary Note 3 for the simulated tomography results). We also found that the relatively long pulses used in the detection sequence act as a filter, suppressing contributions from spins exhibiting the strongest dephasing due to inhomogeneous broadening. As a result, the measurement predominantly probes spins located near the center of the distribution, for which the refocusing procedure is most effective.

**Fig. 4:**
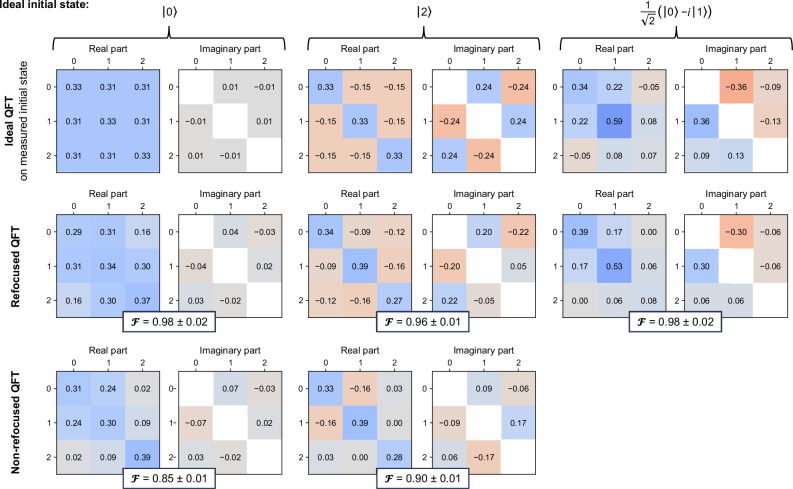
Tomography of the QFT acting on the basis states $\left|0\right\rangle$ and $\left|2\right\rangle$ of the qutrit, and on the superposition state of Fig. 2. We compared the density matrices ${\rho }_{\exp }$ obtained from tomography experiments after implementing the QFT sequences (both refocused and non-refocused) with the density matrix *ρ*_ideal_ obtained by applying *U*_*d*_ to the density matrix of the initial state (*ρ*_0_) extracted from tomography experiments (see Supplementary Fig. 4 for the tomography of *ρ*_0_). For each ${\rho }_{\exp }$ we calculated the fidelity as ${{{\mathcal{F}}}}={{{\rm{Tr}}}}\left(\sqrt{\sqrt{{\rho }_{{{{\rm{ideal}}}}}}{\rho }_{\exp }\sqrt{{\rho }_{{{{\rm{ideal}}}}}}}\right)$, which measures only the performance of pulse sequence implementing the QFT. An error in the order of 0.01 was obtained for all matrix elements from the standard deviation of repeated measurements. The error in the fidelity was obtained by propagating the single-element errors.

We experimentally implemented the QFT, both with the standard (Fig. 3a) and the refocused (Fig. 3c) pulse sequences, on the three basis states. We also tested the refocused sequence with two different superpositions as initial states. Figure 4 shows the tomography (${\rho }_{\exp }$) of the final states after our implementation of the QFT on two of the basis states ($\left|0\right\rangle$, $\left|2\right\rangle$) and the superposition state of Fig. 2. In the same figure, we compare these results with the density matrix *ρ*_ideal_ obtained by applying the ideal QFT operation represented by *U*_*d*_ to the ${\rho }_{\exp }$ obtained from the tomography of the initial states (reported in the Supplementary Note 4). In this case, the quantity ${{{\mathcal{F}}}}={{{\rm{Tr}}}}\left(\sqrt{\sqrt{{\rho }_{{{{\rm{ideal}}}}}}{\rho }_{\exp }\sqrt{{\rho }_{{{{\rm{ideal}}}}}}}\right)$ represents the fidelity of the refocused QFT alone. Similar results for the initial basis state $\left|1\right\rangle$ and superposition $\frac{1}{\sqrt{2}}(\left|1\right\rangle -i\left|2\right\rangle )$ are reported in the Supplementary Note 5. Tomography results after the pristine QFT sequence without refocusing reveal that the populations of the qutrit’s final state are accurately reproduced, whereas the coherences—particularly the two-quanta ones—are substantially attenuated, yielding fidelities ${{{\mathcal{F}}}}\le 0.9$ (e.g., ${{{\mathcal{F}}}}=0.85\pm 0.01$ for the initial state $\left|0\right\rangle$ and ${{{\mathcal{F}}}}=0.90\pm 0.01$ for $\left|2\right\rangle$). Conversely, as one can evince from Fig. 4, the implementation of the refocusing scheme allows for almost a full recovery of all coherences across the density matrix and a consequent improvement of the fidelity ${{{\mathcal{F}}}}\ge 0.96$ (e.g., ${{{\mathcal{F}}}}=0.98\pm 0.02$ for $\left|0\right\rangle$, ${{{\mathcal{F}}}}=0.96\pm 0.01$ for $\left|2\right\rangle$, ${{{\mathcal{F}}}}=0.98\pm 0.02$ for $\frac{1}{\sqrt{2}}(\left|0\right\rangle -i\left|1\right\rangle )$), despite the very long and complex sequence of pulses.

## Discussion

The results demonstrate that we implemented the Quantum Fourier Transform on a molecular spin qudit with a complex sequence of RF pulses, including a refocusing scheme and a full tomography of the final quantum state. The high fidelities resulting from our QFT algorithm, even if still not matching those of well-established platforms like NV centers or transmons^1,49–51^, mark a significant step forward in the field of MSQs. In fact, a direct comparison between these systems and MSQs is not straightforward: the former have benefited from years of studies and optimization, while our proof-of-concept result could still be further improved with optimization techniques. In addition, we note that platforms like NV centers are mainly used as qubits, losing all the advantages of a multi-level computation, while qudits encoded in transmons are affected by the intrinsic noise of their higher excited states. Conversely, MSQs can naturally host a high number of quantum logic states, and our results demonstrate that it is possible to implement complex quantum algorithms on them while retaining precise control over both populations and coherences.

The refocusing scheme designed and implemented in this work has proven to be an effective strategy to suppress on the fly the effects of inhomogeneous broadening in MSQs while implementing the algorithm. We demonstrated experimentally that this scheme mitigates the source of error from inhomogeneous broadening, potentially setting the long characteristic time of pure dephasing in ^173^Yb(trensal) as the time limit for the experiment and opening the door to the implementation of other qudit-based algorithms in our platform. In addition, the refocusing strategy can be easily adapted to other algorithms and to higher qudit dimension *d*, with the time required for a refocusing block scaling as 2*dτ* (where *τ* is the delay between pulses). In this regard, we note that the implementation of the refocusing sequence could be optimized by using a system with more connectivity. For example, refocusing a system with *d* even and loop connectivity—linear connectivity with an additional connection between the edges—requires half the time than a system with linear connectivity. Simulations of our pulse sequences—both refocused and non-refocused—on the ensemble of ^173^Yb(trensal) molecules confirm our experimental results. We simulated the action of our pulse sequences with the experimental parameters, also including the detection scheme. We found that with the non-refocused sequence, coherence is partially lost due to inhomogeneous broadening, as ${T}_{2}^{*}$ for both transitions is much shorter than the sequence duration. In contrast, the ideal simulation of the refocused sequence in the absence of other sources of error completely recovers all coherences. We also employed our simulations to check the effect of introducing a strain in different parameters of the spin Hamiltonian as a source of inhomogeneous broadening. We found the observed ${T}_{2}^{*}$ in the two addressable transitions in this work to be compatible with a strain in the hyperfine couplings being the dominant mechanism (see Supplementary Note 2).

Refocusing strategies for qudits are also exploited in other quantum platforms, where inhomogeneous broadening arises between repetitions of the same experiment in the same quantum system. In transmons^48^ this effect is intrinsic to the system, arising from the additional noisy excited state used to encode the qutrit. In contrast, we implemented a refocusing strategy to mitigate the inhomogeneous broadening coming from working with a large ensemble of molecules. As molecular spins can naturally encode qudits, a more resilient behavior is expected when going to the single molecule limit, since the dominant source of inhomogeneous broadening in our sample is a statistical distribution of hyperfine couplings. However, going beyond ensemble control to achieve single-molecule manipulation and read-out in a scalable platform still represents the primary hurdle to advance MSQ-based computing from proof-of-principle demonstrations towards quantum processors. A promising scalable strategy proposes the integration of MSQs into circuit-QED architectures by, for instance, embedding individual molecules within high-impedance superconducting resonators to achieve strong spin-photon coupling^29^ and dispersive read-out. Alternative control mechanisms through electric fields are also under investigation, which potentially offer a more efficient and spatially-precise single spin-photon coupling^30,31^. Both approaches still pose the challenge of placing a single MSQ in specific points with maximum magnetic or electric coupling to photons in the superconducting circuit. This issue could be overcome via the growth of self-assembling monolayers or 2D molecular arrays on the circuit surface^52,53^, or the directed assembly of nanostructures interfacing MSQs and circuits^54^. Concurrently, implementing two-qudit gates—essential for universal computation—necessitates the development of efficient interaction schemes such as resonant photon-exchange mediated coupling to implement fast two-body operations between molecular units^33^. In this scheme, the ability to tune the resonator frequency via an embedded superconducting quantum interference device (SQUID) is leveraged to move it in and out of resonance with the qudit transition frequencies, enabling a fast switchable coupling. Consider, for example, two non-identical qudits coupled to the same resonator. The photon-mediated qudit-qudit interaction is induced by tuning the resonator alternatively to transitions in each of the qudits. In the case of electro-nuclear systems like ^173^Yb(trensal), with distinct frequencies for all nuclear and electronic spin transitions, this scheme can be implemented by encoding the qudit in the nuclear spin and exciting the electronic spin conditionally on the nuclear spin state. Coupling between electronic spins, which have an enhanced coupling to photons compared to nuclear spins, is then mediated by the resonator to perform two-qudit phase gates. This idea can also be extended to identical qudits by using two coupled tunable resonators. Refocusing protocols like the one introduced in this work could be integrated into these proposed schemes with fast multi-qudit gates, requiring only the refocusing of individual qudits during the idle (i.e., non-coupled) state of the multi-qudit register to implement complex algorithms like the extension of the QFT to multiple qudits. These protocols would still represent a crucial tool to mitigate the effect of other sources of inhomogeneous broadening potentially present even in each individual MSQ, such as the Overhauser field arising from the coupling of the electronic and nuclear spins encoding the qudit with the surrounding nuclear spins of neighbor atoms within the same molecule^55^.

## Methods

### Sample

Tris(2-aminoethyl)amine, salicylaldehyde, acetonitrile, and trifluoromethanesulfonic acid (triflic acid, HOTf) were purchased from commercial sources and used without further purification. ^173^Yb_2_O_3_ was purchased from Neonest AB (Sweden) at 92.7(3)% ^173^Yb enrichment, and Lu_2_O_3_ was purchased from Stanford Advanced Materials at 99.995% purity (REO). ^173^Yb(OTf)_3_ ⋅ 9H_2_O and Lu(OTf)_3_ ⋅ 9H_2_O were prepared by reaction of the corresponding lanthanide oxides with triflic acid, followed by crystallization from ultrapure (M*Ω* ⋅ cm) water, as described for other lanthanides in the literature^56^. In the case of ^173^Yb, the reaction was scaled down to 100 mg of ^173^Yb_2_O_3_ and a stoichiometric amount of triflic acid was used to minimize waste of the isotopically enriched material.

Single crystals of isotopically enriched ^173^Yb(trensal), diluted to a nominal molar concentration of 0.05% in the isostructural Lu(trensal) host, were prepared using a slight modification of the procedure published for Er(trensal)^57^. A solution containing 3.5 ⋅ 10^−4^ mmol ^173^Yb(OTf)_3_ ⋅ 9H_2_O and 0.700 mmol Lu(OTf)_3_ ⋅ 9H_2_O in 15 ml CH_3_CN was slowly added to 205 mg (1.40 mmol, 2 eq.) tris(2-aminoethyl)amine, and the resulting mixture was stirred until dissolved. This solution was placed at the bottom of a glass tube (⌀ ≃ 8mm), layered with an additional 15 ml of acetonitrile and then salicylaldehyde (257 mg, 2.10 mmol, 3 eq.), and the tube was stoppered. Large yellow pencil-shaped crystals grew over 1-2 weeks. Single crystals were isolated by removing the mother liquor, retrieving the crystals on a glass dish, and rinsing with ethanol and diethyl ether.

Small residual crystals from three separate batches of nominal 0.05% ^173^Yb@Lu(trensal) crystallizations were analyzed by inductively coupled plasma mass spectrometry (ICPMS) using an identical procedure for all batches. Between 3.85 and 4.03 mg of ^173^Yb@Lu(trensal) were digested in 1.0 ml of trace analysis nitric acid and diluted to 10.0 ml. A portion of this solution was diluted by half to reach a concentration equivalent to 26.40–27.63 parts-per-billion (ppb) of ^173^Yb. Another portion of the initial solution was diluted by 2000 times (serially 1/20 and 1/100) to afford a concentration equivalent to 53.39–55.89 ppb of Lu. ICPMS results based on a calibration curve, scaled by 0.161/0.927 to reflect the enrichment from 16.1% to 92.7% of ^173^Yb between the calibrants and the sample, found: 25.2688-25.8403 ppb of Yb and 40.4394-58.0067 ppb of Lu, corresponding to an atom percent of 0.0445(6)%–0.0632(9)% Yb@Lu. Calculated errors were propagated from the dilution ratios, neglecting errors in instrument calibration and isotope ratios.

### Pulse generation

The experimental setup consisted of two different pulse generators—a 6 GS/s AWG by Active Technologies, model AWG-5062D, and the HyReSpect spectrometer—that sent pulses to a probehead (an LC circuit with a wide bandwidth) where the sample was located. We chose the orientation of the sample in the probe to have the driving RF magnetic field of the pulses applied along the molecular axis $\widehat{z}$, perpendicular to the static magnetic field. The pulses required for the QFT sequence were generated by the AWG, whereas the Hahn-echo excitation was performed by the spectrometer. The heterodyne-based detection performed by the latter was coherent with its pulse carrier, while the spectrometer and the AWG were mutually incoherent. RF output power was tuned to have a pulse duration of 360 ns for a *π* rotation for pulses in the sequence and 750 ns for the detection. These values were a compromise between leakage—if pulses are too short, they have a wider frequency bandwidth—and inhomogeneity—if pulses are too long, their duration becomes comparable to ${T}_{2}^{*}$. Leakage to other transitions outside the qutrit subspace was mitigated by the bandwidth of the excitation pulses and by the probe-head bandwidth, which included only the relevant transition frequencies *f*_01_ and *f*_12_. The detected in-phase and quadrature components of the echo were analyzed with a classical Fourier Transform (FT) and a phase correction. The experimental value is the amplitude of the FT at the resonance frequency.

### Qutrit state tomography

We performed a complete tomography of the final state after the implementation of the QFT through a set of measurable quantities *q* from which the density matrix can be reconstructed. Our observable was the magnetization of the molecular spin qudit along the $\widehat{z}$ axis, which generated the RF signal detected by the spectrometer. Thus, our tomography procedure translated each *q* into the amplitude of this signal: each *q* value was extracted one at a time by implementing the QFT, storing the specific *q* as a population difference between two states $\left|\mu \right\rangle$ and $\left|\nu \right\rangle$ with additional pulses (see Table 1), and then detecting this difference with a Hahn-echo sequence incoherent with the QFT sequence. This process was averaged 1000 times for each *q*. The fact that AWG and spectrometer were mutually incoherent ensured that any echoes generated by the interplay between pulses sent by different instruments were averaged out on signal accumulation. Thus, only the echo generated by the spectrometer pulses was detected, proportional to the quantity *q* stored as a population difference. The set of quantities *q* required to perform a full qutrit tomography is reported in Table 1, along with the pulse sequences that transform each *q* into a population difference.

**Table 1:** The eight quantities *q* required for a full tomography of the 3 × 3 density matrix describing the qutrit state, with the pulses needed to transform each *q* into a population difference and the frequency (transition) in which this difference is detected with a Hahn-echo sequence

Tomography scheme		
*q*	Pulses (*θ*, *ϕ*, *f*_*μν*_)	Detection (*f*_*μν*_)
${\rho }_{00} - {\rho }_{11}$	–	*f*_01_
${\rho }_{11} - {\rho }_{22}$	–	*f*_12_
$2\,{{{\rm{Re}}}}({\rho }_{01})$	(*π*/2, − *π*/2, *f*_01_)	*f*_01_
$2\,{{{\rm{Im}}}}({\rho }_{01})$	(*π*/2, *π*, *f*_01_)	*f*_01_
$2\,{{{\rm{Re}}}}({\rho }_{12})$	(*π*/2, − *π*/2, *f*_12_)	*f*_12_
$2\,{{{\rm{Im}}}}({\rho }_{12})$	(*π*/2, *π*, *f*_12_)	*f*_12_
$2\,{{{\rm{Re}}}}({\rho }_{02})$	(*π*, − *π*/2, *f*_12_),	*f*_01_
	then (*π*, − *π*/2, *f*_01_),	
	then (*π*/2, *π*/2, *f*_01_)	
$2\,{{{\rm{Im}}}}({\rho }_{02})$	(*π*, − *π*/2, *f*_12_),	*f*_01_
	then (*π*, *π*, *f*_01_),	
	then (*π*/2, 0, *f*_01_)	

Notation for pulses is (*θ*, *ϕ*, *f*_*μν*_), where *θ* is the tilt angle, *ϕ* the phase of the carrier signal and *f*_*μν*_ the carrier frequency.

In the case of the refocused QFT, the set of *q* values was extracted at the moment of the last refocused state (before the last *π* pulse, see Fig. 3c). After 3 refocusing blocks of six pulses each, both sequences A and B introduced in this work to implement the QFT need one last *π* pulse to complete the algorithm. However, this pulse just swaps two basis states, and we designed the two sequences to have different states involved in the swap ($\left|0\right\rangle$ and $\left|1\right\rangle$ in sequence A, $\left|1\right\rangle$ and $\left|2\right\rangle$ in sequence B). In this way, each sequence allows a detection of different density matrix elements on a refocused state. Note that this swapping between states can easily be reverted during the actual implementation of either of the two sequences, once the QFT is integrated as part of a larger algorithm. Matrix elements measured using both sequences A and B were averaged, mitigating any possible offset error.

### Pseudo-pure state generation

The initial state of the ensemble before any manipulation is a thermal state, that is, a completely mixed state with each eigenstate of the Hamiltonian of Eq.(1) having its Boltzmann population at temperature *T* = 1.4 K. With a suitable manipulation process, a subspace of the full density matrix can be converted into a pseudo-pure state with equal elements in the diagonal except for a specific eigenstate. In our case, we are interested in the subspace spanned by the three states we labeled $\left|0\right\rangle$, $\left|1\right\rangle$ and $\left|2\right\rangle$. In this subspace, the pseudo-pure state is generated with a *π*/2-pulse with frequency *f*_12_, giving equal population to states $\left|1\right\rangle$ and $\left|2\right\rangle$, followed by a wait much longer than *T*_2_ that lets the coherence between them decay. The resulting density matrix in the $\left\{\left|0\right\rangle,\left|1\right\rangle,\left|2\right\rangle \right\}$ subspace has therefore the form

$$\begin{array}{ll}{\rho }_{0-2}^{{\prime} } &=\left(\begin{array}{rcl}{p}_{0} & 0 & 0\\ 0 & \frac{{p}_{1}+{p}_{2}}{2} & 0\\ 0 & 0 & \frac{{p}_{1}+{p}_{2}}{2}\end{array}\right)=\\ &=\left(\begin{array}{rcl}{\alpha }_{0}+\frac{{p}_{1}+{p}_{2}}{2} & 0 & 0\\ 0 & \frac{{p}_{1}+{p}_{2}}{2} & 0\\ 0 & 0 & \frac{{p}_{1}+{p}_{2}}{2}\end{array}\right)=\\ &=\,{\alpha }_{0}\left|0\right\rangle \left\langle 0\right|\,+\,\frac{{p}_{1}+{p}_{2}}{2}\,{\mathbb{1}},\end{array}$$

with *α*_0_ = *p*_0_ − (*p*_1_ + *p*_2_)/2 and *p*_*η*_ (*η* = 0, 1, 2) the initial Boltzmann population of the energy states. Except for a normalization factor (*α*_0_), this state is equivalent to the pure density matrix ${\rho }_{0-2}=\left|0\right\rangle \left\langle 0\right|$. Indeed, the part of ${\rho }_{0-2}^{{\prime} }$ proportional to the identity in the considered subspace (${\mathbb{1}}$) does not produce any signal in our experiment. It is also worth noting that we can work with a pseudo-pure state restricted to a 3-state subspace of the full 12-state qudit thanks to the symmetry properties of the static Hamiltonian of Eq.(1). When the static magnetic field is applied along the $\widehat{x}$ axis, Eq.(1) is invariant under a *π* rotation around this same axis. Conversely, our observable is the magnetization of the molecular spin qudit along $\widehat{z}$, which has then zeros all along the diagonal if written in the eigenbasis of the static Hamiltonian. Thus, only coherences generated by our pulses on the two addressable transitions can be detected: outside the 3-state subspace, the contribution is zero as the trace of the product of a diagonal matrix (describing the thermal populations of the other 9 states) and a matrix with all zeros in the diagonal (the observable) is zero.

### Normalization of tomography experiments

The measurement of the echo on transition $\left|\mu \right\rangle \leftrightarrow \left|\nu \right\rangle$ after a Hahn-echo sequence with frequency *f*_*μν*_, starting from thermal equilibrium, yields an echo intensity ${I}_{\mu \nu }^{({{{\rm{eq}}}})}={C}_{\mu \nu }\left[{p}_{\mu }^{({{{\rm{eq}}}})}-{p}_{\nu }^{({{{\rm{eq}}}})}\right]$. Here, *C*_*μν*_ is a constant proportional to the matrix element of the magnetization along $\widehat{z}$ between the two states, and ${p}_{\mu }^{({{{\rm{eq}}}})}$ is the population of state $\left|\mu \right\rangle$ in thermal equilibrium. When we generate the pseudo-pure state, the same transition produces an echo intensity ${I}_{\mu \nu }^{({{{\rm{pur}}}})}={C}_{\mu \nu }\left[{p}_{\mu }^{({{{\rm{pur}}}})}-{p}_{\nu }^{({{{\rm{pur}}}})}\right]$, now with ${I}_{12}^{({{{\rm{pur}}}})}\propto {p}_{1}^{({{{\rm{pur}}}})}-{p}_{2}^{({{{\rm{pur}}}})}\simeq 0$. After manipulating the pseudo-pure state and converting a quantity *q* into a population difference, the value of *q* is obtained from the corresponding echo intensity as

$$q={p}_{\mu }-{p}_{\nu }=\frac{1}{\alpha }\frac{{I}_{\mu \nu }}{{I}_{\mu \nu }^{({{{\rm{eq}}}})}}\left[{p}_{\mu }^{({{{\rm{eq}}}})}-{p}_{\nu }^{({{{\rm{eq}}}})}\right],$$

which depends on a normalization factor *α*. This factor, obtained from the measurement of ${I}_{01}^{({{{\rm{pur}}}})}$ and ${I}_{12}^{({{{\rm{pur}}}})}$, ensures that the trace of the density matrix representing the pseudo-pure state—and also of the density matrices representing the manipulation of this state—equals 1 and that all elements in its diagonal are positive. For an ideal pseudo-pure state with ${I}_{12}^{({{{\rm{pur}}}})}=0$, the normalization factor is

$$\alpha=\frac{{I}_{01}^{({{{\rm{pur}}}})}}{{I}_{01}^{({{{\rm{eq}}}})}}\left[{p}_{0}^{({{{\rm{eq}}}})}-{p}_{1}^{({{{\rm{eq}}}})}\right]=\left[{p}_{0}^{({{{\rm{pur}}}})}-{p}_{1}^{({{{\rm{pur}}}})}\right]={\alpha }_{0}\,.$$

A small ${I}_{12}^{({{{\rm{pur}}}})}$ leads to a correction in the normalization factor,

$$\alpha={\alpha }_{0}+\frac{1+3\,\,{{{\rm{sgn}}}}\,(\epsilon )}{2}\,| \epsilon | \left[{p}_{1}^{({{{\rm{eq}}}})}-{p}_{2}^{({{{\rm{eq}}}})}\right]\,,$$

with $\epsilon={I}_{12}^{({{{\rm{pur}}}})}/{I}_{12}^{({{{\rm{eq}}}})}$. Thus, all measurements are normalized by measuring the echo intensities of the thermal equilibrium and pseudo-pure states for both transitions, plus a temperature measurement (*T* = 1.4 K) to compute the population differences in thermal equilibrium: ${p}_{0}^{({{{\rm{eq}}}})}-{p}_{1}^{({{{\rm{eq}}}})}=7.705\cdot 1{0}^{-4}$, ${p}_{1}^{({{{\rm{eq}}}})}-{p}_{2}^{({{{\rm{eq}}}})}=8.343\cdot 1{0}^{-4}$.

### Simulations

Each of our simulations starts from a single electronic spin-nuclear spin pair described by the static Hamiltonian of Eq.(1) with the parameters and experimental conditions (*B*_0_ = 0.2 T along the molecular $\widehat{x}$ axis) reported in the main text. On top of the free evolution under Eq.(1), state manipulation via a sequence of driving pulses was represented by a time-dependent Zeeman term with an oscillating magnetic field along $\widehat{z}$ (perpendicular to **B**_0_). We then introduced inhomogeneous broadening as a normal distribution in a parameter (generically labeled *P* here) of Eq.(1) with values around a mean *P*_0_ (the value reported in the main text), discretised taking equally spaced values of *P* with weights *w*(*P*). The best parameter choice and standard deviation of the distribution were optimized to fit the experimental width of the echoes, which we generated by: i) running the simulation for a spin with a given *P* and recording its magnetization along $\widehat{z}$, proportional to the signal picked by the coil of the probe, in the time window where the echo was expected, and ii) averaging the result for all values of *P* in the distribution. We found that experiment and simulation were compatible only if *P* was the hyperfine coupling (see Supplementary Note 2). The optimized distribution was used to simulate the effect of all QFT sequences, with the resulting echoes processed as the experimental ones to complete the state tomography.

## Supplementary information

Supplementary Information

Transparent Peer Review file

## Data Availability

The data that support the findings of this study are available in Zenodo with the identifier 10.5281/zenodo.18741561^58^.
