# Supplementary material for: Implementing the Quantum Fourier Transform on a molecular qudit with full refocusing and state tomography
Source: Nat Commun. 2026 May 7;17:6217. doi: 10.1038/s41467-026-72390-z (PMC13369187; doi:10.1038/s41467-026-72390-z)
Supplement: Supplementary file 1 — Supplementary Information [file 41467_2026_72390_MOESM1_ESM.pdf]

SUPPLEMENTARY INFORMATION
Implementing the Quantum Fourier Transform on a molecular qudit with full refocusing and state tomography

Marcos Rubín-Osanz[1], Laura Bersani[1], Simone Chicco[1], Giuseppe Allodi[1], Roberto De Renzi[1], Athanasios Mavromagoulos[2], Michael D. Roy[2], Stergios Piligkos[2*], Elena Garlatti[1,3*], Stefano Carretta[1,3*]
[1]*Dipartimento di Scienze Matematiche, Fisiche e Informatiche, Università di Parma and UdR Parma, INSTM, I-43124 Parma, Italy.*
[2]*Department of Chemistry, University of Copenhagen, DK-2100 Copenhagen, Denmark.*
[3]*INFN, Sezione di Milano-Bicocca, gruppo collegato di Parma, I-43124 Parma, Italy.*

*Corresponding authors. Email: stefano.carretta@unipr.it, elena.garlatti@unipr.it, piligkos@chem.ku.dk

I. SUPPLEMENTARY NOTE 1: SAMPLE CHARACTERIZATION

Supplementary Fig. 1a shows the spectrum (black dots with solid line) of ^{173}Yb(trensal) within the probe bandwidth for $B_0 = 0.2$ T. Vertical lines mark the expected transitions with resonant frequencies between 270 and 410 MHz, including: i) the two addressable nuclear spin transitions between the three states defining our qutrit subspace (solid lines), with frequencies f_{01} and f_{12}, and ii) the rest of nuclear spin transitions outside this subspace (grey dashed lines). Driving pulses with f_{01} or f_{12} could lead to an unwanted manipulation of transitions with neighbour frequencies, thus we specifically designed a probe with a limited bandwidth (quality factor $Q \sim 20$) to exclude all transitions outside the qutrit subspace.

Supplementary Fig. 1b shows the result of thermal spin lattice relaxation recovery experiments on transitions $|0\rangle \leftrightarrow |1\rangle$ and $|1\rangle \leftrightarrow |2\rangle$. The initial condition was obtained with a train of pulses equipopulating the two states, followed by a Hahn echo detection ($\frac{\pi}{2} - \pi$ sequence) with a variable τ delay, during which the system start to recover. We obtained relaxation times of at least 2 ms for the fastest relaxing spins, two orders of magnitude longer than the duration of our sequences implementing the QFT. Conversely, the waiting time $t \gg T_2$ to let the coherence between states $|1\rangle$ and $|2\rangle$ decay during the pseudo-pure state generation procedure was long enough to introduce a slight error relative to their equipopulation due to spin-lattice relaxation. We compensated this error by fine tuning the duration of the equipopulating pulse, ideally a $\pi/2$ pulse without relaxation, until we minimized the population difference between $|1\rangle$ and $|2\rangle$.

For the measurement of the phase memory time T_2 (reported in Fig. 1c of the main text), a simple echo sequence (with equal pulses $\frac{2\pi}{3} - \frac{2\pi}{3}$, to maximize the signal while irradiating the same frequency band) was implemented, varying the interval between the two pulses. Since the shape of the decay is Gaussian, the extracted T_2 was defined as the value at which the signal amplitude reached $1/e$ of its maximum value.

Rabi oscillations (reported in Fig. 1d of the main text) were sampled by sending a variable-θ pulse, whose duration was finely increased, followed by a refocusing π pulse in a spin-echo sequence.

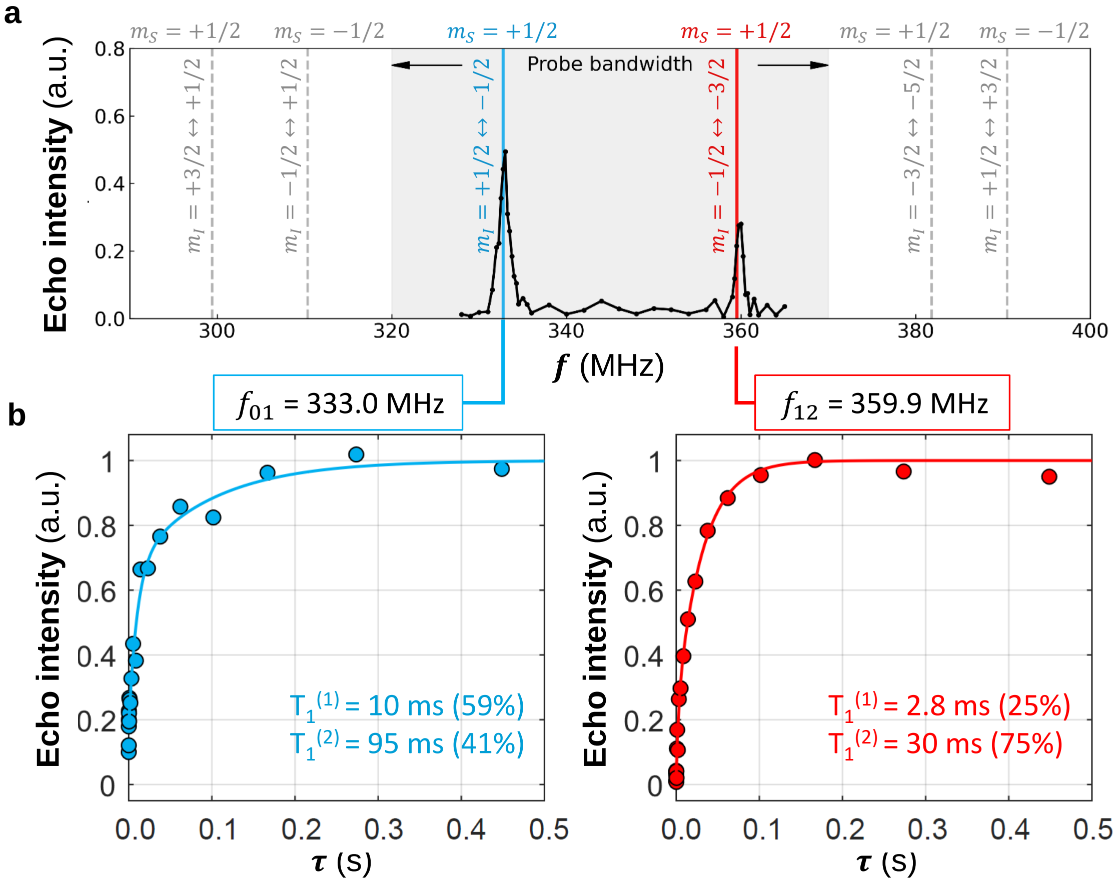

Supplementary Figure 1. Spectrum and relaxation of the two addressable qutrit transitions. a Measured spectrum (black dots with solid line) within the probe bandwidth (shaded area). The two expected transition frequencies f_{01} and f_{12} in this range are shown with solid lines. Transition frequencies outside the probe bandwidth are shown as grey dashed lines. **b** *Left*: relaxation of the $|0\rangle \leftrightarrow |1\rangle$ transition, with frequency $f_{01} = 333.0$ MHz. *Right*: relaxation of the $|1\rangle \leftrightarrow |2\rangle$ transition, with frequency $f_{12} = 359.9$ MHz. In both cases, relaxation is best fit as a bi-exponential decay, with characteristic times $T_1^{(1)}$ (fast) and $T_1^{(2)}$ (slow), which is not unusual for a multilevel system with different possible relaxation pathways. The relative weight of both contributions is indicated in %. Error bars are of the order of scatter dimension; they were obtained by applying to the noise the same analysis we applied for the data (see Methods) and taking the root mean square.

II. SUPPLEMENTARY NOTE 2: SIMULATION AND ORIGIN OF INHOMOGENEOUS BROADENING

In order to have a good estimate of the effect of inhomogeneous broadening in the results, we simulated the Hahn-echo sequence for each transition and compared the result with the measured thermal equilibrium state echoes (Supplementary Fig. 2). We introduced inhomogeneous broadening as a collection of molecular spins described by the Hamitonian of Eq.(1) in the main text, each with different values of a specific parameter of the Hamiltonian. The resulting magnetization traces for the different spins were averaged according to the weights of a normal distribution of the chosen parameter centered in its previously reported value (see more in Methods in the main text). Indeed, this generated an echo after a $\pi/2$-π sequence of pulses, with the duration of the echo depending on the effect of the chosen parameter on the states involved in the transition. We found that the origin that better describes the relative duration of the echoes in the two addressed transitions is not a distribution in the parameters of the electronic Zeeman term but a distribution in the hyperfine couplings, with $|\sigma_A/A| = 0.15\%$. In fact, introducing a distribution in the Zeeman term leads to a significantly shorter echo duration for transition $|1\rangle \leftrightarrow |2\rangle$, as $\left|\frac{df_{12}}{dB}\right| > \left|\frac{df_{01}}{dB}\right|$, which we do not observe. Thus, in our simulations we only included a distribution in the hyperfine couplings to reproduce our experimental data.

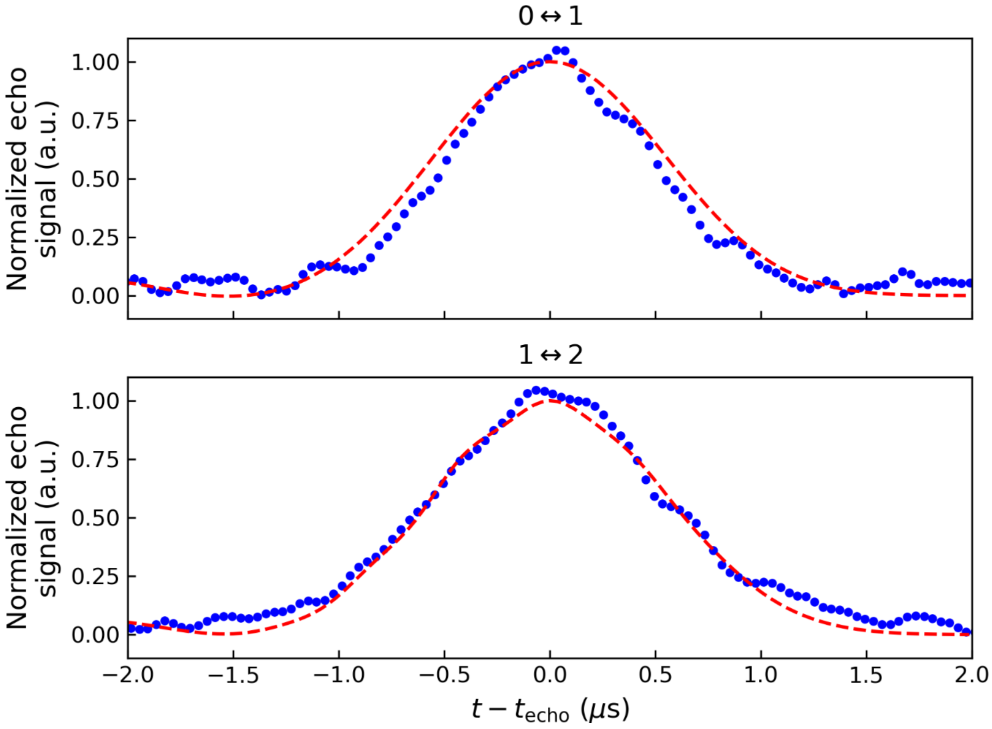

Supplementary Figure 2. Thermal echo in the two addressable transitions. Comparison between the measured (blue dots) and simulated (red dashed-lines) echoes. *Top*: transition $|0\rangle \leftrightarrow |1\rangle$. *Bottom*: transition $|1\rangle \leftrightarrow |2\rangle$.

The experimental apparatus may also introduce inhomogeneity, but a possible error in the manipulation of the state (that might be corrected with a dedicated sequence) does not affect the free evolution of the state nor the echo formation. The effect of non-instantaneous pulses is included in the simulation instead. We found that the relatively long pulses used by the spectrometer in the Hahn-echo detection (750 ns for a π-pulse, compared to 360 ns with the AWG in the QFT sequence) broaden the measured echo, which leads to an overestimation by eye of the characteristic decay time due to inhomogeneity (T_2^*). All these considerations—and the extracted parameters—depict how the inhomogeneous broadening was implemented in all simulations (including those reported in the Supplementary Note 3).

III. SUPPLEMENTARY NOTE 3: SIMULATION OF QFT SEQUENCES AND TOMOGRAPHY EXPERIMENTS

We simulated the non-refocused and refocused QFT pulse sequences implementing the QFT using a distribution with the same strain $|\sigma_A/A|$ determined in the Supplementary Note 2 to describe inhomogeneous broadening. After each sequence, we also simulated the pulses that transfer the different q to population differences, as well as their incoherent Hahn-echo detection with the spectrometer. Supplementary Fig. 3 shows the result of these simulations with the three basis states of the qutrit as initial states. The simulation of the non-refocused sequence yields an attenuation of coherences, markedly on the coherence between $|1\rangle$ and $|2\rangle$ and the two-quanta coherence between $|0\rangle$ and $|2\rangle$, as observed in the experiment. These results suggest inhomogeneous broadening as the main source of error in the implementation of the QFT with the non-refocused sequence. Conversely, the simulation of the refocused sequence gives an almost perfect implementation of the QFT (in the absence of other sources of error apart from inhomogeneous broadening).

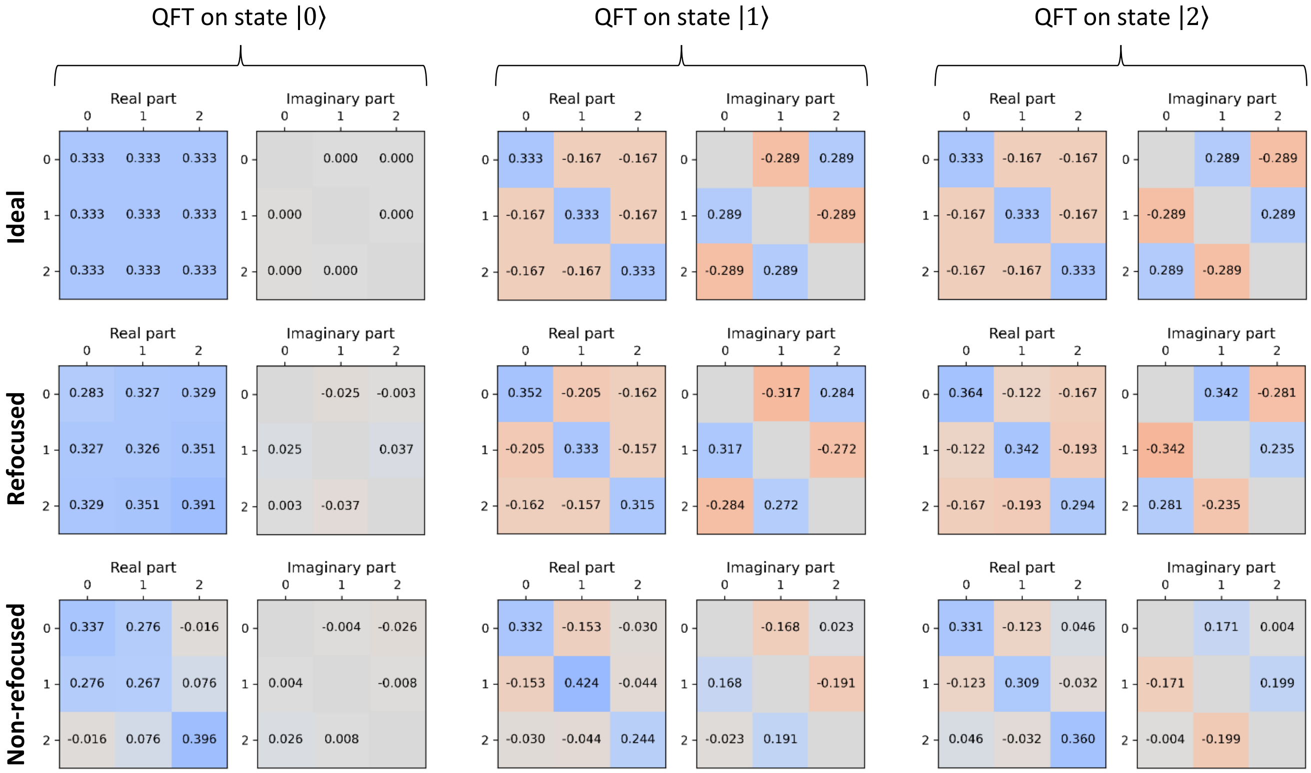

Supplementary Figure 3. Comparison between the ideal QFT and the simulation of the refocused and non-refocused implementation on initial states $|1\rangle$ and $\frac{1}{\sqrt{2}}(|1\rangle - i|2\rangle)$. Simulations were carried out with experimental parameters, including only inhomogeneous broadening as a source of error. The implementation of the QFT is almost perfect with the refocused sequence, while the non-refocused sequence yields final states with attenuated coherences.

IV. SUPPLEMENTARY NOTE 4: EXPERIMENTAL DATA OF THE TOMOGRAPHY OF THE INITIAL STATES

We report in Supplementary Fig. 4 the tomography of four out of the five initial states used to test our QFT sequences: $|0\rangle$, $|1\rangle$, $|2\rangle$ and the superposition state $\frac{1}{\sqrt{2}}(|1\rangle - i\,|2\rangle)$. The tomography for the fifth initial state, $\frac{1}{\sqrt{2}}(|0\rangle - i\,|1\rangle)$, is shown in Fig. 2b of the main text. For each initial state, we compare the density matrix of the ideal state (ρ_{ideal}) with the density matrix we recover from tomography experiments after the experimental manipulation of the pseudo-pure state (ρ_{exp}), obtaining fidelities $\mathcal{F} = \text{Tr}\left(\sqrt{\sqrt{\rho_{\text{ideal}}}\rho_{\text{exp}}\sqrt{\rho_{\text{ideal}}}}\right)$ between 0.95 and 0.98 for the initial state generation step. The error in $\mathcal{F}$ was estimated from the noise in the echo traces following the analysis of Figs. 1 and 2c in the main text.

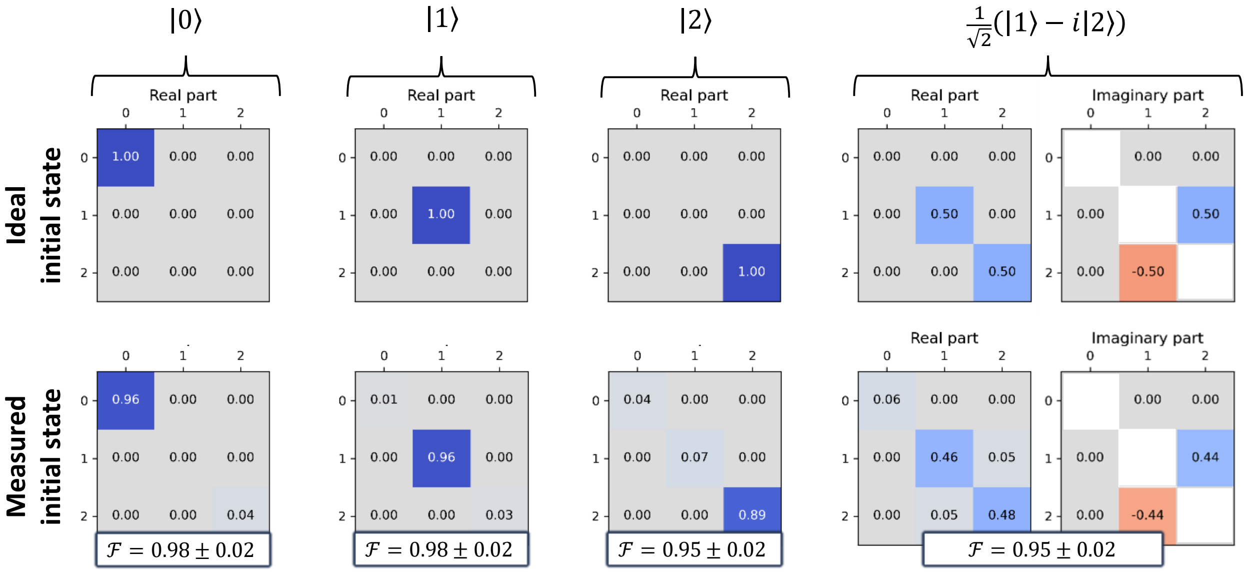

Supplementary Figure 4. Tomography of the initial states for the QFT. Initial states were generated from the pseudo-pure state, here shown as the measured initial state $|0\rangle$, with sequences combining $\theta = \pi$ and $\theta = \pi/2$ pulses on the two addressable transitions. The tomography of the fifth initial state $\frac{1}{\sqrt{2}}(|0\rangle - i\,|1\rangle)$ can be found in Fig. 2b of the main text.

V. SUPPLEMENTARY NOTE 5: EXPERIMENTAL DATA OF THE TOMOGRAPHY OF THE STATES AFTER THE IMPLEMENTATION OF THE QFT

Here we report those measurements from our full set of tomography data after the implementation of the QFT that were not included in the main text. Supplementary Fig. 5 compares the non-refocused and refocused QFT implementations for the third basis state, $|1\rangle$. The fidelity of the experimental implementation of the QFT is calculated as $\mathcal{F} = \mathrm{Tr}\left(\sqrt{\sqrt{\rho_{\mathrm{ideal}}}\rho_{\mathrm{exp}}\sqrt{\rho_{\mathrm{ideal}}}}\right)$, where ρ_{ideal} is the density matrix obtained by applying U_d to the initial state obtained from a tomography experiment (see Supplementary Fig. 4), and ρ_{exp} is the density matrix obtained from the tomography of the state after sending the QFT sequence (refocused or non-refocused). With the non-refocused sequence we obtain a fidelity $\mathcal{F} = 0.90 \pm 0.01$, which improves to $\mathcal{F} = 0.95 \pm 0.02$ using the refocused QFT sequence. Figure S5 also shows the the result of the refocused QFT on the superposition state $\frac{1}{\sqrt{2}}\left(|1\rangle - i\,|2\rangle\right)$, with a fidelity $\mathcal{F} = 0.97 \pm 0.01$.

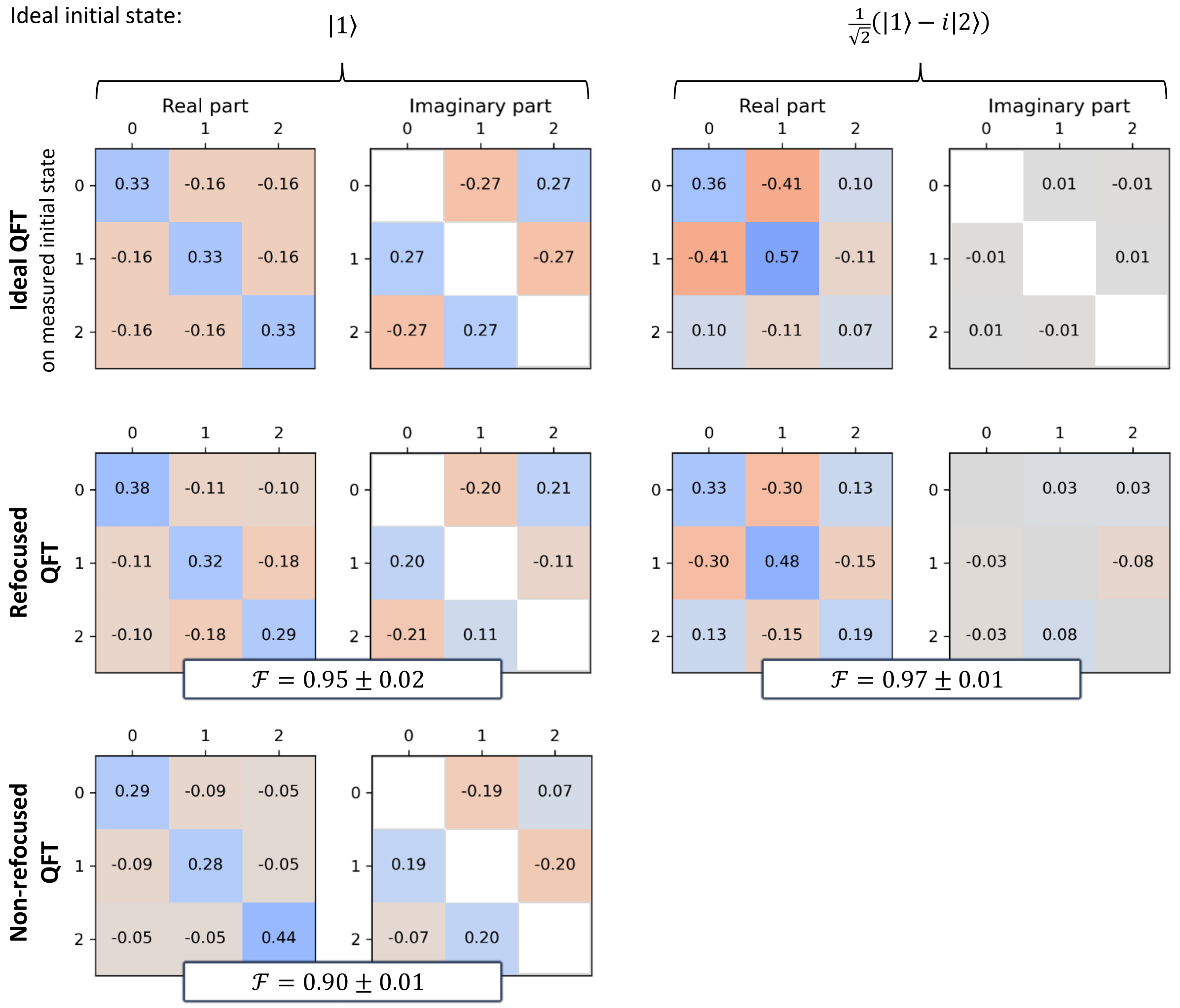

Supplementary Figure 5. Comparison between the ideal QFT (U_d) on the experimental initial states and its experimental implementation (refocused and non-refocused) on initial states $|1\rangle$ and $\frac{1}{\sqrt{2}}\left(|1\rangle - i\,|2\rangle\right)$. First row was obtained by applying U_d to the tomography of the initial states shown in the bottom row of Supplementary Fig. 4.
