# Supplementary material for: Implementing the Quantum Fourier Transform on a molecular qudit with full refocusing and state tomography
Source: Nat Commun. 2026 May 7;17:6217. doi: 10.1038/s41467-026-72390-z (PMC13369187; doi:10.1038/s41467-026-72390-z)
Supplement: Supplementary file 2 — Transparent Peer Review file [file 41467_2026_72390_MOESM2_ESM.pdf]

nature portfolio

Peer Review File

Implementing the Quantum Fourier Transform on a molecular qudit with full refocusing and state tomography

Corresponding Author: Professor Stefano Carretta

This file contains all reviewer reports in order by version, followed by all author rebuttals in order by version.

Version 0:

Reviewer comments:

Reviewer #1

(Remarks to the Author)
The authors report on the experimental implementation of the QFT algorithm on a three-level molecular spin qudit system. The experiment is performed using a home-built NMR spectrometer with broadband capabilities, which allows addressing all three states spanning a 50 MHz range. Due to the fast T2* decay of the system, which consists of an ensemble of molecules in a crystal, implementing QFT requires to integrate a refocusing sequence into the overall algorithm. Refocusing procedures are anyway well-known and commonly applied in NMR. Detection of the successful exploitation of QFT is done using a complete state tomography. The results are interesting and well presented, and sets an important milestone for the application of molecular spins as qubits. Nevertheless, there are a few points that would be worth to address before its publication.

1) The description of experimental details of the molecule used for the experiment is completely missing. The authors should mention at least the synthetical procedure, source and purity of the chemicals, degree of the isotopic enrichment, and purity of the crystal that was used.
2) A description of the sample preparation and experimental conditions should be reported.
3) It would be useful to mention also the procedure used for the simulation of the ideal density matrix and for the refocusing sequence.
4) Fig.1: the black line on top of the Rabi oscillations is not mentioned in the caption.
5) What is the frequency of the other transitions? Could it be possible that they are also inducing other transitions besides the 01 and 12?
6) Fig.3: what are A and B sequences? The caption indicates to look in the Methods, but it is not really explained what is their role. The authors should explain it better in the main manuscript.
7) It remains unclear to me how the authors can be convinced that the main source of inhomogeneous broadening are strains of the hyperfine. Couldn't it come from the experimental apparatus?
8) It would be useful to compare the results obtained here with what is possible to achieve using transmon qubits. In particular, the use of such a three-level system provides any advantage for the implementation of QFT? Are the fidelities obtained comparable with those of transmons? And compared to NV center in diamonds?
9) Pag7, row450: pn does not appear in the equation.
10) There are several typos in the Supporting Information, I invite the authors to give a second read to it.

Reviewer #2

(Remarks to the Author)
I have read the work by the authors "Implementation of the Quantum Fourier Transform on a molecular qudit with full refocusing and state tomography". They experimentally implement the QFT in a molecular spin qudit of Yb-Trensal and introduce a refocusing scheme to overcome the T_2^* dephasing, produced by dipolar interactions in the diluted system. Overall, I think that this work is very interesting and demonstrates the possibility to implement the QFT in an experimental platform that has some appealing features. Their results are relevant for the community working on quantum computation with molecular spins, but also for experimental groups using different architectures, which can benefit from the refocusing algorithm on the fly that has been proposed here. I also think that the manuscript is well-written and clear, and that their

experimental results are well supported by the numerical simulations made in parallel. For all these reasons I recommend publication with just some minor modifications/suggestions:

1) In the introduction, the authors mention that molecular spins can be scaled up by means of integrating them in circuit QED architectures. I think this is really hard due to the seemingly small coupling to single molecules. I think this statement in the introduction should be more cautious or supported by additional references.

2) In the text the authors claim that the Gaussian shape of the decay is due to the dipolar interactions. Then, should I expect that changing dilution will only affect the width of the Gaussian, but not the overall shape?

3) I am not completely sure what the authors mean in the paragraph discussing the initial preparation. In particular, the statement about the decomposition of the density matrix as a pure state plus a identity matrix. I think this paragraph should be better explained.

4) The authors have demonstrated the implementation of the QFT in a qutrit subspace of the molecule, but it seems to me that due to dephasing, refocusing will not be enough if they try to implement it in larger subspaces, involving more levels. This seems like a series constrain to scale-up the QFT. Is this correct? If that is the case, this should probably be mentioned, together with ways to maybe overcome this issue in the future.

Reviewer #3

(Remarks to the Author)
Title: Implementation of the Quantum Fourier Transform on a molecular qudit with full refocusing and state tomography

In their manuscript ‘Implementation of the Quantum Fourier Transfrom on a molecular qudit with full refocusing and state tomography’, authors report an experimental demonstration of a qudit-version of Quantum Fourier Transform using hyperfine-coupled spin qudits in well-designed molecular magnets. The implementation with a tailored full-refocusing technique also enhanced the overall fidelity of the scheme. The authors further provide full state tomography and quantitatively confirm the overall fidelities.
This work addresses the physical implementation of a qudit-version of Quantum Fourier Transform, which is key ingredient for universal quantum information processing. In addition, the manuscript is clearly written and the experimental results technically sound. The results provided in the manuscripts represent unique experimental demonstration of a quantum information processing scheme based on molecular spin qudits, which has potential to be expended to other platforms, and offers resource-efficient design for future universal quantum computers.
While these points of novelty are worth enough to justify publication on Nature Communications, there are several concerns regarding explanation and interpretation of technical details, which are currently presented without giving enough details to readers. For these reasons, below points should be addressed before recommending publications:

Comment 1: The authors define the Quantum Fourier Transform (QFT) based on Eq.3 in the manuscript, which corresponds to the definition of QFT within a single-qudit Hilbert space. However, for the practical usefulness in quantum information processing, a controlled-operation of this unitary (Ud) gate is essential. Indeed, the overall Hamiltonian given in Eq.1 implies that spectral frequency addressing of all individual transitions is available, and controlled transition from electron spin (S=1/2) to hyperfine coupled nuclear spin (I=5/2) is possible. In other words, the two transitions at 333 MHz and 359 MHz already correspond to controlled operations that act only on the nuclear spin when the electron spin is in the +1/2 state. However, the authors should explicitly describe these features in the manuscript to claim a practically meaningful QFT.

Comment 2: As discussed in Comment 1, the entire QFT process in qudits consists of controlled-Ud gates between multiple qudits. In this manuscript, the authors only mention QFT gates within single qudit, however, there should be strategies to extend this Ud gate operations to multiple qudits. Since the overall experimental scheme in this work based on molecular spin qudits (MSQ), at least a brief roadmap for realizing multi-qudit QFT gates within MSQ systems is required.

Comment3 : The authors offer a dedicate full-refocusing pulse sequence that suppress inhomogeneous broadening effects in a spin ensemble system (T2*) and keep the coherence for a relatively long time within T2 and/or T1 limitations of the qudits. While this refocusing scheme works well with the proof-of concept level for presented this qutrit system, there should be a discussion on how this number of refocusing pulses scales with the qudit dimension (d) and number of qudits (n). It would also be useful to note together the single pulse (or gate) fidelities required to achieve reasonable QFT process fidelities.

Comment 4 : In page 3, the statement “This demonstrates our coherent control of the qutrit state beyond Rabi experiments, with a gate fidelity F … = 0.97 ± 0.02.” This sounds like the fidelity of state preparation and tomography, rather than the single-gate operation fidelity. I wonder whether the single gate fidelities are better than this value, and whether this can be enhanced with additional pulse sequences such as BB1 sequences (https://doi.org/10.1006/jmra.1994.1159).

Comment 5. Molecular spin qudits (MSQ) can be a promising platform for quantum information processing, and the authors implemented the QFT successfully, however, these results are confirmed using a spin ensemble. How can these results be further improved toward the level of single-spin manipulation and readout?

Comment 6: In the Supplementary Information Chapter 2, the authors claim that inhomogeneous broadening mostly comes from a strain-induced distribution of the hyperfine coupling (A), rather than from a distribution of the Zeeman Splitting. I think

record of the inhomogeneous broadening according to the external B0 field sweep will give more clear evidence. Is there corresponding data?

Version 1:

Reviewer comments:

Reviewer #1

(Remarks to the Author)
The authors have provided exhaustive answers to all my questions and addressed the issues I raised. I am satisfied with the current status of the manuscript and recommend its publication in Nature Communications without further modification.

Reviewer #2

(Remarks to the Author)
The authors have successfully addressed all my concerns and I think that the new version of the manuscript has improved. Therefore, I recommend its publication in its current form.

Reviewer #3

(Remarks to the Author)
The authors have satisfactorily addressed my concerns and comments. I therefore recommend the manuscript for publication in Nature Communications.

Open Access This Peer Review File is licensed under a Creative Commons Attribution 4.0 International License, which permits use, sharing, adaptation, distribution and reproduction in any medium or format, as long as you give appropriate credit to the original author(s) and the source, provide a link to the Creative Commons license, and indicate if changes were made.
In cases where reviewers are anonymous, credit should be given to 'Anonymous Referee' and the source.
The images or other third party material in this Peer Review File are included in the article's Creative Commons license, unless indicated otherwise in a credit line to the material. If material is not included in the article's Creative Commons license and your intended use is not permitted by statutory regulation or exceeds the permitted use, you will need to obtain permission directly from the copyright holder.
To view a copy of this license, visit https://creativecommons.org/licenses/by/4.0/

Reviewer #1 (Remarks to the Author):

The authors report on the experimental implementation of the QFT algorithm on a three-level molecular spin qudit system. The experiment is performed using a home-built NMR spectrometer with broadband capabilities, which allows addressing all three states spanning a 50 MHz range. Due to the fast T2* decay of the system, which consists of an ensemble of molecules in a crystal, implementing QFT requires to integrate a refocusing sequence into the overall algorithm. Refocusing procedures are anyway well-known and commonly applied in NMR. Detection of the successful exploitation of QFT is done using a complete state tomography. The results are interesting and well presented, and sets an important milestone for the application of molecular spins as qubits. Nevertheless, there are a few points that would be worth to address before its publication.

We thank the Reviewer for the positive report and for stating that our work "sets an important milestone for the application of molecular spins as qubits". We also thank them for the careful and critical reading of the manuscript and their insightful comments and questions, which we addressed below.

1. **The description of experimental details of the molecule used for the experiment is completely missing. The authors should mention at least the synthetical procedure, source and purity of the chemicals, degree of the isotopic enrichment, and purity of the crystal that was used.**

   We agree with the Reviewer in that the source purity of the chemicals, the degree of the isotopic enrichment and the purity of the crystal are specific to the sample with which the experiment was carried out, therefore they should be included in the text. We have now added these details, along with the synthetical procedure used, to the updated *Sample* section in *Methods*:

   "Tris(2-aminoethyl)amine, salicylaldehyde, acetonitrile, and trifluoromethanesulfonic acid (triflic acid, HOTf) were purchased from commercial sources and used without further purification. $^{173}Yb_2O_3$ was purchased from Neonest AB (Sweden) at 92.7(3)% ^{173}Yb enrichment and Lu_2O_3 was purchased from Stanford Advanced Materials at 99.995% purity (REO). $^{173}Yb(OTf)_3{\cdot}9H_2O$ and $Lu(OTf)_3{\cdot}9H_2O$ were prepared by reaction of the corresponding lanthanide oxides with triflic acid, followed by crystallization from ultrapure (MΩ·cm) water, as described for other lanthanides in the literature[56]. In the case of ^{173}Yb, the reaction was scaled down to 100 mg of $^{173}Yb_2O_3$ and stoichiometric amount of triflic acid was used to minimize waste of the isotopically enriched material.

   Single crystals of isotopically enriched ^{173}Yb(trensal), diluted to a nominal molar concentration of 0.05% in the isostructural Lu(trensal) host, were prepared using a slight modification of the procedure published for Er(trensal)[57]. A solution containing $3.5{\cdot}10^{-4}$ mmol $^{173}Yb(OTf)_3{\cdot}9H_2O$ and 0.700 mmol $Lu(OTf)_3{\cdot}9H_2O$ in 15 ml CH_3CN was slowly added to 205 mg (1.40 mmol, 2 eq.) tris(2-aminoethyl)amine, and the resulting mixture was stirred until dissolved. This solution was placed in the bottom of a glass tube (⌀≃ 8 mm), layered with an additional 15 ml of acetonitrile and then salicylaldehyde (257 mg, 2.10 mmol, 3 eq.), and the tube was stoppered. Large yellow pencil-shaped crystals grew over 1-2 weeks. Single crystals were isolated by removing the mother liquor, retrieving the crystals on a glass dish, and rinsing with ethanol and diethyl ether.

Small residual crystals from three separate batches of nominal 0.05% ^{173}Yb@Lu(trensal) crystallizations were analysed by inductively coupled plasma mass spectrometry (ICPMS) using an identical procedure for all batches. Between 3.85 and 4.03 mg of 173Yb@Lu(trensal) were digested in 1.0 ml trace analysis nitric acid and diluted to 10.0 ml. A portion of this solution was diluted by half to reach a concentration equivalent to 26.40-27.63 parts-per-billion (ppb) of ^{173}Yb. Another portion of the initial solution was diluted by 2000 times (serially 1/20 and 1/100) to afford a concentration equivalent to 53.39-55.89 ppb of Lu. ICPMS results based on a calibration curve, scaled by 0.161/0.927 to reflect the enrichment from 16.1% to 92.7% of ^{173}Yb between the calibrants and the sample, found: 25.2688-25.8403 ppb of Yb and 40.4394-58.0067 ppb of Lu, corresponding to an atom percent of 0.0445(6)% - 0.0632(9)% Yb@Lu. Calculated errors were propagated from the dilution ratios, neglecting errors in instrument calibration and isotope ratios."

In this text we added two new references:

- [56] Vesborg, P. C. K.; Chorkendorff, I.; Brock-Nannestad, T.; Dethlefsen, J. R.; Bendix, J. Note: Simple means for selective removal of the 365 nm line from the Hg spectrum using Dy. *Rev. Sci. Instrum.* **82**, 096102 (2011).
  Link: https://doi.org/10.1063/1.3639128
- [57] Pedersen, K. S. et al. Modifying the properties of 4f single-ion magnets by peripheral ligand functionalisation. *Chem. Sci.* **5**, 1650-1660 (2014).
  Link: https://doi.org/10.1039/C3SC53044B

2. A description of the sample preparation and experimental conditions should be reported.

Sample preparation was relatively straightforward, but the Reviewer is right to say that it should be reported nonetheless. The ^{173}Yb@Lu(trensal) crystal was inserted inside the coil of the probe, with the correct alignment, using a custom non-magnetic sample holder. This information can be found now in the *Results* section - *Characterization and calibration of the qudit*:

"For the implementation of the QFT algorithm, the single crystal was inserted inside the coil of a custom multi-frequency probe and placed at the centre of the bore of a 9 T superconducting magnet. The crystal was aligned with a custom non-magnetic sample holder to set the C_3 axis of the ^{173}Yb(trensal) molecules perpendicular to the magnetic field B_0 inside the magnet bore ($B_0 \parallel x$)."

Experimental conditions were already reported in the main text. In the revised main text they can be found now in line 148 (B_0=0.2 T) and line 170 (T = 1.4 K).

3. It would be useful to mention also the procedure used for the simulation of the ideal density matrix and for the refocusing sequence.

We thank the Reviewer for this suggestion. We agree that giving more details about the procedure used for our simulations will further help the understanding of our results. Our simulations start from the free evolution of a single electronic spin-nuclear spin, representing the ^{173}Yb(trensal) molecule, described by the static Hamiltonian of Eq.(1).

The radiofrequency pulse sequences to control and readout the system are introduced via a time-dependent Zeeman term with an oscillating magnetic field perpendicular to the static field. The inhomogeneity is introduced by executing the simulation on an ensemble of qudits with slightly different static Hamiltonian parameters. The choice of parameter that varies from spin to spin and the width of its distribution around a mean value determines the amplitude and shape of the echo that is obtained by combining the output of all simulations. Following the advice from the Reviewer, we have added a new section to the *Methods*, titled *Simulations,* that addresses the points mentioned above:

"Each of our simulations starts from *a single electronic spin-nuclear spin pair described by the static Hamiltonian of Eq.(1) with the parameters and experimental conditions (B_0 = 0.2 T along the molecular x axis) reported in the main text. On top of the free evolution under Eq.(1), state manipulation via a sequence of driving pulses was represented by a time-dependent Zeeman term with an oscillating magnetic field along z (perpendicular to B_0). We then introduced inhomogeneous broadening as a normal distribution in a parameter (generically labelled P here) of Eq.(1) with values around a mean P_0 (the value reported in the main text), discretised taking equally spaced values of P with weights w(P). The best parameter choice and standard deviation of the distribution were optimised to fit the experimental width of the echoes, which we generated by: i) running the simulation for a spin with a given P and recording the magnetization along z, proportional to the signal picked by the coil of the probe, in the time window where the echo was expected, and ii) averaging the result for all values of P in the distribution. We found that experiment and simulation were compatible only if P was the hyperfine coupling (see Fig. S2 in the Supplementary Information). The optimized distribution was used to simulate the effect of all QFT sequences, with the resulting echoes processed as the experimental ones to complete the state tomography."*

4. **Fig.1: the black line on top of the Rabi oscillations is not mentioned in the caption.**

   We thank the Reviewer for pointing it out. The black line is a fit of the Rabi oscillations generated with the spectrometer, using a pulse power that gives a π-pulse duration of 750 ns. We have now updated the caption of Figure 1:

   "Black solid line is the fit of the Rabi oscillations, which gives a π-pulse duration of 750 ns for the spectrometer."

5. **What is the frequency of the other transitions? Could it be possible that they are also inducing other transitions besides the 01 and 12?**

   Indeed, as the Reviewer suggests, in the absence of the probe bandwidth there may be an unwanted driving (leakage) of transitions outside the qutrit subspace, comparable to the already small intra-subspace leakage. For this reason, we designed the probe to have a limited bandwidth (quality factor Q ~ 20) that excludes these neighbour frequencies. This is shown in a new figure (Fig. S1a, reproduced below) in Supplementary Note 1, where we also added a discussion about leakage and the probe bandwidth:

"Figure S1a shows the spectrum (black dots with solid line) of ^{173}Yb(trensal) within the probe bandwidth for B_0 = 0.2 T. Vertical lines mark the expected transitions with resonant frequencies between 270 and 410 MHz, including: i) the two addressable nuclear spin transitions between the three states defining our qutrit subspace (solid lines), with frequencies f_{01} and f_{12}, and ii) the rest of nuclear spin transitions outside this subspace (grey dashed lines). Driving pulses with f_{01} or f_{12} could lead to an unwanted manipulation of transitions with neighbour frequencies, thus we specifically designed a probe with a limited bandwidth (quality factor Q ~ 20) to exclude all transitions outside the qutrit subspace."

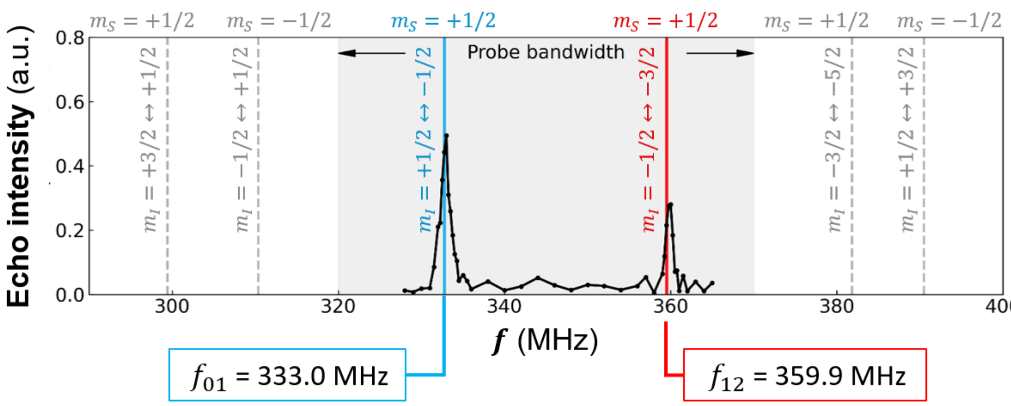

In addition, we explicitly refer now both to Fig. S1a and to the Supplementary Note 1 in the main text (*Results - Characterization and calibration of the qudit*):

"A comparison between the measured spectrum, the probe bandwidth and the frequencies of neighbour transitions is reported in the Supplementary Information (SI)."

6. **Fig.3: what are A and B sequences? The caption indicates to look in the Methods, but it is not really explained what is their role. The authors should explain it better in the main manuscript.**

We thank the Reviewer for raising this point, allowing us to better explain a subtle aspect of our refocusing procedure. We indeed want to perform the tomography of the state of the qudit after the QFT on a refocused state, requiring 3 refocusing blocks of six pulses each. The implementation of the U_d operation of Eq.(3) would require an additional π pulse, whose effects on the tomography result is only to swap the basis states in which the density matrix elements are measured, which can be easily taken into account when reconstructing the density matrix. To do this, we designed two complementary sequences (labelled A and B) that give access to coherences and population differences between different states in the density matrix before the last π pulse. Once the QFT is integrated as part of a larger algorithm, it could be implemented with any of the full A or B sequences. We updated the main manuscript trying to better address all these points, specifically in the *Qutrit state tomography* section in *Methods*:

"After 3 refocusing blocks of six pulses each, both sequences A and B introduced in this work to implement the QFT need one last π pulse to complete the algorithm. However, this pulse just swaps two basis states, and we designed the two sequences to have

different states involved in the swap (|0⟩ and |1⟩ in sequence A, |1⟩ and |2⟩ in sequence B). In this way, each sequence allows a detection of different density matrix elements on a refocused state. […] Matrix elements measured using both sequences A and B were averaged, mitigating any possible offset error."

In addition, we tried to be more clear in the introduction of sequences A and B in the caption of Fig. 3 before referring to *Methods*:

"Detection was performed on a refocused state, before the last π pulse that only introduces a swap. Both sequences A and B implement the QFT, each one allowing for a simple detection of different density matrix elements (see Methods)."

7. **It remains unclear to me how the authors can be convinced that the main source of inhomogeneous broadening are strains of the hyperfine. Couldn't it come from the experimental apparatus?**

We will try to summarize here first our ideas about the origin of inhomogeneous broadening for the Reviewer. If there were no inhomogeneity coming from a distribution of a parameter in Eq.(1), the inhomogeneity in the evolution of the system would enter only through the time-dependent interaction Hamiltonian (for example, an inhomogeneity in the driving magnetic field B_1 felt by each molecule). This is indeed a possible source of error in the manipulation of the state, which can be mitigated with dedicated sequences like the BB1 sequence mentioned by Reviewer 3. Thus, the experimental apparatus has an effect on the manipulation pulses which may result in a spread of the ensemble, and can change the shape of the echo, as we discuss later. However, it cannot be by itself the source of the echo, which arises from the free evolution of the ensemble and it is the result of a loss of coherence that can be recovered with a suitable sequence of pulses. Since during the free evolution there is no external manipulation, the inhomogeneity describing this loss of coherence comes from the parameters of the system and not from the external apparatus. In particular, the time dependence of the signal detected by the spectrometer is governed only by Eq.(1). In this regard, a minimal model for the echo is obtained choosing a suitable parameter (hyperfine coupling) in Eq.(1) to introduce inhomogeneity so that simulations are compatible with the echoes that are observed in the experiment.

We do agree with the Reviewer in that the experimental apparatus does play a role in the shape of the echo generated by an inhomogeneity in Eq.(1): i) it may cause an additional spread of the ensemble during the manipulation pulses due to a B_1 inhomogeneity, as mentioned above, ii) a small probe bandwidth may distort the echo, iii) the bandwidth of the manipulation pulses may also distort the echo. In our experiment, the latter effect is particularly relevant, as the duration of the pulses sent by the spectrometer are comparable to T_2^*. This effect is included in our simulations, and the extent of the distribution in the hyperfine coupling was determined including it.

In summary, we agree with the Rewiewer in that our original wording was probably too strong. The claim in the *Discussion* section states now that our results are <u>compatible</u> with a simple model that introduces inhomogeneous broadening only through the hyperfine coupling, without ever stating that it is its only source:

"We found the observed T_2^* in the two addressable transitions in this work to be compatible with a strain in the hyperfine couplings being the dominant mechanism (see SI)."

Several highlighted new sentences can be found now in the Supplementary Note 2 addressing details of both this question and question 3.

As a final remark, in case the Reviewer meant the static field B_0 generated by the magnet as a possible source of inhomogeneity due to the experimental apparatus (instead of the Zeeman coupling of the spin system), we want to note that the stability and homogeneity of B_0 in our magnets has been tested in previous experiments with NMR reference samples.

8. **It would be useful to compare the results obtained here with what is possible to achieve using transmon qubits. In particular, the use of such a three-level system provides any advantage for the implementation of QFT? Are the fidelities obtained comparable with those of transmons? And compared to NV center in diamonds?**

We thank the Referee for this insightful question, which gives us the opportunity to better contextualize our results with respect to other leading solid-state quantum platforms. A comparison between all these platforms is not straightforward. The latest results in transmon qubits report single-qubit gate and two-qubits gate fidelities over 99.9% and 99%, respectively (see Reference 1). The computational advantage of using them as qudits has been also recently explored through their coupling with resonators. In particular, the QFT algorithm for *d*=3 has been implemented with a fidelity of 0.992 (new reference 49). These results obviously benefit of years of studies and optimization of transmons, even if in most transmon-based architectures their weak anharmonicity generally limits the use of higher excited states to auxiliary roles for gate implementation.

Similarly, NV centers single-qubit can achieve record fidelities up to 99.999% and two-qubit gate fidelities of 99.93%, using an optimized control (new reference 50). However, this platform is limited to a qutrit encoding, and the intrinsic nature of vacancies/defects poses limitations for scalability. In addition, QFT-related protocols realized with NV centers-based platforms are optimized for sensing performance rather than for benchmarking universal quantum algorithms with full process or state tomography. (new reference 51). Thus, in this case a direct comparison with our QFT implementation is more difficult.

Conversely, molecular nanomagnets are more suitable to encode qudits than the aforementioned platforms, since they naturally provide an engineerable multi-level energy spectrum and thus a well-defined, spectrally resolvable computational space. From this perspective, our result constitutes a proof-of-principle demonstration of the potential of molecular spin qudits for quantum algorithms. The fidelity values obtained in this work primarily reflects the early-stage nature of coherent control in molecular spin platforms, rather than a fundamental limitation of the qudit approach itself, and further improvements could be obtained with optimization techniques (e.g. pulse shaping to minimize leakage outside of the desired quantum subspace). Indeed, the use of the additional degrees of freedom of qudits can largely increase the power of quantum logic for many applications, with respect to qubit-based architectures. For instance, qudit encoding can significantly reduce the number of two-body gates, reduce

the quantum circuit depth and thus improve the implementation of quantum algorithms and quantum simulations. Moreover, by encoding a protected qubit into a single multilevel object, quantum error correction could be implemented without the large overhead of resources required by qubit-based codes.

We have updated the *Discussion* section to include a succinct version of these remarks:

"The high fidelities resulting from our QFT algorithm, even if still not matching those of well established platforms like NV centres or transmons[1,49-51], mark a significant step forward in the field of MSQs. In fact, a direct comparison between these systems and MSQs is not straightforward: the former benefit of years of studies and optimization, while our proof-of-concept result could still be further improved with optimization techniques. In addition, we note that platforms like NV centres are mainly used as qubits, losing all the advantages of a multi-level computation, while qudits encoded in transmons are affected by the intrinsic noise of their higher excited states. Conversely, MSQs can naturally host a high number of quantum logic states […]"

In this text we added three new references:

- [49] Champion, E., et al., Efficient control of a transmon qudit using effective spin-7/2 rotations. *Phys. Rev. X* **15**, 021096 (2025).
  Link: https://journals.aps.org/prx/abstract/10.1103/vbh4-lysv
  [50] Bartling, H. et al., Universal high-fidelity quantum gates for spin qubits in diamond. *Phys. Rev. Appl.* **23**, 034052 (2025).
  Link: https://link.aps.org/doi/10.1103/PhysRevApplied.23.034052.
  [51] Vorobyov, V. et al. Quantum Fourier Transform for nanoscale quantum sensing. *npj Quantum Information* **7**, 124 (2021).
  Link: https://www.nature.com/articles/s41534-021-00463-6

9. Pag7, row450: pn does not appear in the equation.

Here η is just a subscript for *p*. To avoid confusion, this is stated explicitly in the new first paragraph of the *Pseudo-pure state generation* section in *Methods* that we wrote for the answer to question 3 by Reviewer 2.

10. There are several typos in the Supporting Information, I invite the authors to give a second read to it.

We thank the Reviewer for carefully proofreading the manuscript and identifying typos. We found and corrected the following misspellings and typographical errors (indicating the line in the revised version): "*an -> a*" in line 20, removed duplicated "*a*" in line 33, sorted % which did not add up to 100% due to bad rounding in Fig. S1b (right panel), "*suggests -> suggest*" in line 63, "*of -> of the*" in line 80. We hope those were all.

<u>Reviewer #2 (Remarks to the Author)</u>:

I have read the work by the authors "Implementation of the Quantum Fourier Transform on a molecular qudit with full refocusing and state tomography". They experimentally implement the QFT in a molecular spin qudit of Yb-Trensal and introduce a refocusing scheme to overcome the T_2^* dephasing, produced by dipolar interactions in the diluted system. Overall, I think that this work is very interesting and demonstrates the possibility to implement the QFT in an experimental platform that has some appealing features. Their results are relevant for the community working on quantum computation with molecular spins, but also for experimental groups using different architectures, which can benefit from the refocusing algorithm on the fly that has been proposed here. I also think that the manuscript is well-written and clear, and that their experimental results are well supported by the numerical simulations made in parallel. For all these reasons I recommend publication with just some minor modifications/suggestions:

We are grateful to the Reviewer for the careful reading of the manuscript and for recommending its publication on Nature Communications. We also thank them for the useful remarks raised, which are discussed point by point below.

1. **In the introduction, the authors mention that molecular spins can be scaled up by means of integrating them in circuit QED architectures. I think this is really hard due to the seemingly small coupling to single molecules. I think this statement in the introduction should be more cautious or supported by additional references.**

   We agree with the Reviewer in that one of the current challenges of integrating single molecules in circuit QED architectures is the strength of the coupling through the Zeeman term (coupling to magnetic fields), and that this strategy for scaling up should be presented to the reader in a more cautious (yet still promising) way. In this regard, we included a reference to a very recent work available in arXiv (new reference 29) that reports a single spin-photon coupling of 100 kHz in such a circuit QED architecture. In addition, we note there are groups in the field of molecular spin qudits working in an alternative control mechanism through electric fields, which potentially offer a more efficient and spatially-precise single spin-photon coupling (new reference 30). In this case, the molecular design has to be tailored to obtain a useful coupling to electric fields (new reference 31).

   We have expanded the comment on scalability through circuit QED architectures in the *Introduction*, presented now as a more cautious (as the Reviewer asks for) short-term perspective with the state-of-the-art references mentioned above and not as a current feature of MSQ (such as long coherence times or multi-level energy spectrum):

   "The design of MQS with a nuclear spin—naturally encoding a qudit—coupled to the effective molecular electronic spin also provides a pathway for scalability through the coupling of the latter to the magnetic[29] and electric[30,31] fields of superconducting resonators in circuit quantum electrodynamics architectures[32,33]."

   In this text we added three new references:

   - [29] Rubín-Osanz, M. et al. Optimizing magnetic coupling in lumped element superconducting resonators for molecular spin qubits (2025).

Link: https://doi.org/10.48550/arXiv.2511.00857

- [30] Liu, J. et al. Quantum coherent spin–electric control in a molecular nanomagnet at clock transitions. *Nat. Phys.* **17**, 1205–1209 (2021).
  Link: https://doi.org/10.1038/s41567-021-01355-4
- [31] Vaganov, M. V. et al. Chemical tuning of quantum spin–electric coupling in molecular magnets. *Nat. Chem.* **17**, 1903–1909 (2025).
  Link: https://doi.org/10.1038/s41557-025-01926-5

There are also some minor changes in other parts of the second paragraph of the *Introduction*, they are all highlighted in the revised main text.

2. **In the text the authors claim that the Gaussian shape of the decay is due to the dipolar interactions. Then, should I expect that changing dilution will only affect the width of the Gaussian, but not the overall shape?**

We thank the Reviewer for this observation, as we forgot to specify that we were referring to the **nuclear** dipolar interaction that induces decoherence in the MQS through the nuclear spin bath (e.g. interactions between ^{1}H and ^{14}N in the molecular structure). The Gaussian shape of the decay is typical of decoherence processes induced mainly by nuclear spins degrees of freedom. The magnetic dilution instead, affects only the inter-molecular electronic dipolar interaction (between the electronic magnetic moments of Yb ions). At higher concentrations of magnetic ions than the one used in the experiment, the molecular electronic dipolar interaction would become more and more relevant, shortening T_2 and distorting the Gaussian shape into an exponential decay. We have now specified "nuclear dipolar interaction" at the end of *Characterization and calibration of the qudit* section:

"The Gaussian shape of the decay of coherences, typical of decoherence processes induced mainly by the degrees of freedom of the nuclear spin bath, is expected if nuclear dipolar interactions are the dominant dephasing mechanism."

3. **I am not completely sure what the authors mean in the paragraph discussing the initial preparation. In particular, the statement about the decomposition of the density matrix as a pure state plus an identity matrix. I think this paragraph should be better explained.**

We appreciate the feedback from the Reviewer about the clarity of this point in the text. We have now rewritten all the paragraphs concerning the pseudo-pure state generation trying to describe the process as clear as possible. See highlighted parts in the last paragraph in the *Results – Coherent control and qutrit state tomography* section and the completely new first paragraph in *Methods – Pseudo-pure state generation*:

"The initial state of the ensemble before any manipulation is a thermal state, that is, a completely mixed state with each eigenstate of the Hamiltonian of Eq.(1) having its Boltzmann population at temperature $T = 1.4$ K. With a suitable manipulation process, a subspace of the full density matrix can be converted into a pseudo-pure state, with equal elements in the diagonal except for a specific eigenstate. In our case, we are interested in the subspace spanned by the three states we labelled $|0\rangle$, $|1\rangle$ and $|2\rangle$. In this subspace, the pseudo-pure state is generated with a $\pi/2$-pulse with frequency f_{12}, giving

equal population to states |1⟩ and |2⟩, followed by a wait much longer than T_2 that lets the coherence between them decay. The resulting density matrix in the {|0⟩, |1⟩, |2⟩} subspace has therefore the form:

$$\rho_{\frac{\pi}{2}} = \begin{pmatrix} p_0 & 0 & 0 \\ 0 & \frac{p_1+p_2}{2} & 0 \\ 0 & 0 & \frac{p_1+p_2}{2} \end{pmatrix} = \begin{pmatrix} \alpha_0 + \frac{p_1+p_2}{2} & 0 & 0 \\ 0 & \frac{p_1+p_2}{2} & 0 \\ 0 & 0 & \frac{p_1+p_2}{2} \end{pmatrix} = \alpha_0|0\rangle\langle 0| + \frac{p_1+p_2}{2}\mathbb{I}$$

with $\alpha_0 = p_0 - (p_1 + p_2)/2$ and p_η (η = 0, 1, 2) the initial Boltzmann population of the energy states."

4. **The authors have demonstrated the implementation of the QFT in a qutrit subspace of the molecule, but it seems to me that due to dephasing, refocusing will not be enough if they try to implement it in larger subspaces, involving more levels. This seems like a series constrain to scale-up the QFT. Is this correct? If that is the case, this should probably be mentioned, together with ways to maybe overcome this issue in the future.**

We understand the Reviewer's concern about the scaling of the refocusing procedure, which was not stated in the text. The refocusing block can be generalized for $d > 3$, with the depth of the block (time needed to implement it) scaling with $2d$ (for linear connectivity) or d (for loop or all-to-all connectivity). The number of such blocks needed to perform a refocused implementation of an algorithm depends also on the choice of decomposition of U_d in planar rotations and phase gates. In this regard, we are currently working on adapting known decomposition strategies for which the number of blocks would scale with $2d-3$ or even d. This would be still within the experimental limitations due to pure dephasing (T_2) if d is not too large. In addition, we also estimated the expected fidelity of the QFT as a function of the single pulse fidelity for different dimension d:

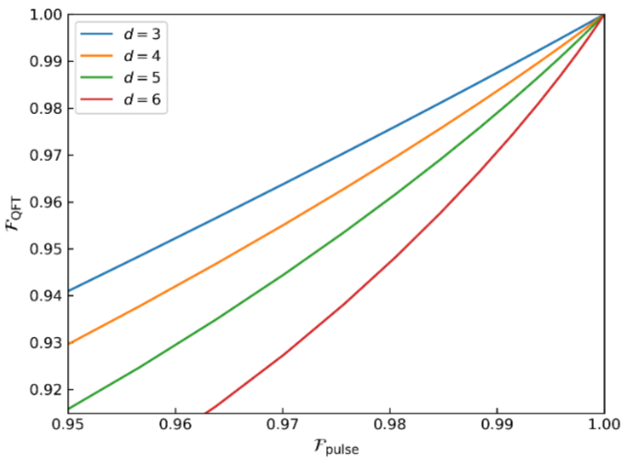

Here we assumed a single pulse error affecting only coherences (which is similar to an error due to pure dephasing). This estimation predicts over 0.92 for the QFT in dimension d with the same sample and under the same experimental conditions (same T_2).

We updated the *Discussion* in the main text in order to explicitly state the scaling of the refocusing block duration with *d*:

"[…] with the time required for a refocusing block scaling as 2dτ (where τ is the delay between pulses). […] For example, refocusing a system with d even and loop connectivity—linear connectivity with an additional connection between the edges—requires half the time than a system with linear connectivity."

<u>Reviewer #3 (Remarks to the Author)</u>:

In their manuscript 'Implementation of the Quantum Fourier Transform on a molecular qudit with full refocusing and state tomography', authors report an experimental demonstration of a qudit-version of Quantum Fourier Transform using hyperfine-coupled spin qudits in well-designed molecular magnets. The implementation with a tailored full-refocusing technique also enhanced the overall fidelity of the scheme. The authors further provide full state tomography and quantitatively confirm the overall fidelities. This work addresses the physical implementation of a qudit-version of Quantum Fourier Transform, which is key ingredient for universal quantum information processing. In addition, the manuscript is clearly written and the experimental results technically sound. The results provided in the manuscripts represent unique experimental demonstration of a quantum information processing scheme based on molecular spin qudits, which has potential to be expended to other platforms, and offers resource-efficient design for future universal quantum computers. While these points of novelty are worth enough to justify publication on Nature Communications, there are several concerns regarding explanation and interpretation of technical details, which are currently presented without giving enough details to readers. For these reasons, below points should be addressed before recommending publications:

We thank the Reviewer for the positive comments about the novelty, the significance and the soundness of the results of our manuscript. We also thank them for their very useful remarks and suggestions, which will improve the presentation of our work. Please find below point-by-point answers and subsequent changes and additions in the text.

1. **The authors define the Quantum Fourier Transform (QFT) based on Eq.3 in the manuscript, which corresponds to the definition of QFT within a single-qudit Hilbert space. However, for the practical usefulness in quantum information processing, a controlled-operation of this unitary (Ud) gate is essential. Indeed, the overall Hamiltonian given in Eq.1 implies that spectral frequency addressing of all individual transitions is available, and controlled transition from electron spin (S=1/2) to hyperfine coupled nuclear spin (I=5/2) is possible. In other words, the two transitions at 333 MHz and 359 MHz already correspond to controlled operations that act only on the nuclear spin when the electron spin is in the +1/2 state. However, the authors should explicitly describe these features in the manuscript to claim a practically meaningful QFT.**

   We thank the Reviewer for raising this crucial point. Indeed, as the Reviewer suggests, the implementation of the QFT in this work is conditional on the electron spin state. This is possible in ^{173}Yb(trensal) thanks to the distinct nuclear transition frequencies of the

m_S = +1/2 and m_S = -1/2 manifolds. We thank the Reviewer for pointing it out, and a statement about it can be found now in the *"Characterization and calibration of the qudit"* section:

"Any operation limited to this subspace can be understood also as a controlled gate acting on the nuclear spin state only if m_S = +1/2. This is possible in ^{173}Yb(trensal) thanks to the distinct nuclear transition frequencies of the m_S = +1/2 and m_S = -1/2 manifolds."

In addition, a figure was added to Supplementary Note 1 (Fig. S1a) showing the probe bandwidth and the measured spectrum within it compared to the expected transition frequencies based on previous characterization experiments of ^{173}Yb(trensal). This figure, reproduced below, shows also how the same nuclear transition has a different frequency depending on the state of the electronic spin (with a difference of at least 20 MHz).

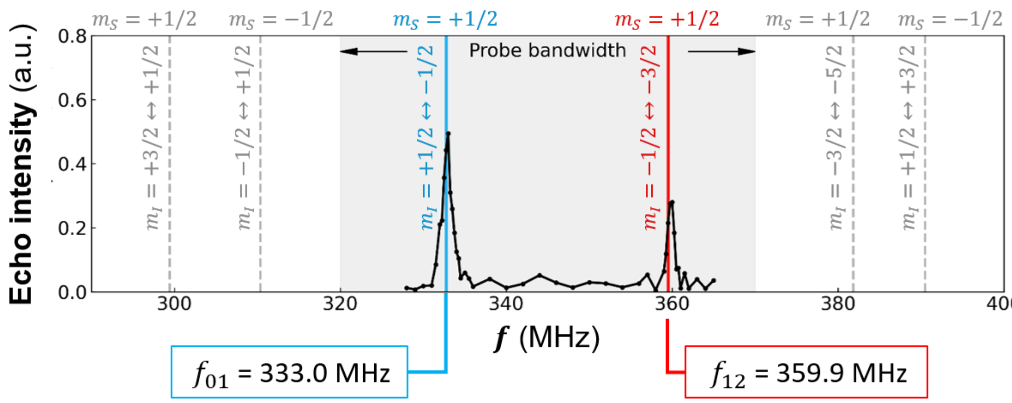

2. **As discussed in Comment 1, the entire QFT process in qudits consists of controlled-Ud gates between multiple qudits. In this manuscript, the authors only mention QFT gates within single qudit, however, there should be strategies to extend this Ud gate operations to multiple qudits. Since the overall experimental scheme in this work based on molecular spin qudits (MSQ), at least a brief roadmap for realizing multi-qudit QFT gates within MSQ systems is required.**

We agree with the Reviewer on the importance of discussing strategies to extend the QFT to multiple qudits, in order to give further perspective to our results. The single qudit gate U_d in this work has to be combined with two-qudit gates to implement a multi-qudit QFT. In this regard, we updated the *Discussion* section with possible ways to scale up with MSQs and create a multi-qudit register, including two-qudit gates. As the new text deals with several related questions (2, 3 and 5) posed by this Reviewer, we reproduce below the parts related to this specific question:

"A promising scalable strategy proposes the integration of MSQs into circuit-QED architectures by, for instance, embedding individual molecules within high-impedance superconducting resonators […] Concurrently, implementing two-qudit gates—essential for universal computation—necessitates the development of efficient interaction schemes such as resonant photon-exchange mediated coupling to implement fast two-body operations between molecular units[33]. In this scheme, the ability to tune the resonator frequency via an embedded superconducting quantum interference device

(SQUID) is leveraged to move it in and out of resonance with the qudit transition frequencies, enabling a fast switchable coupling. Consider, for example, two non-identical qudits coupled to the same resonator. The photon-mediated qudit-qudit interaction is induced by tuning the resonator alternatively to transitions in each of the qudits. In the case of electro-nuclear systems like ^{173}Yb(trensal), with distinct frequencies for all nuclear and electronic spin transitions, this scheme can be implemented by encoding the qudit in the nuclear spin and exciting the electronic spin conditionally on the nuclear spin state. Coupling between electronic spins, which have an enhanced coupling to photons compared to nuclear spins, is then mediated by the resonator to perform two-qudit phase gates. This idea can be also extended to identical qudits by using two coupled tunable resonators. […] to implement complex algorithms like the extension of the QFT to multiple qudits."

We hope the Reviewer is fine with our choice to merge the answers to questions 2, 3 and 5 in the revised manuscript, we thought this solution provided a more readable and smooth *Discussion* section.

3. **The authors offer a dedicate full-refocusing pulse sequence that suppress inhomogeneous broadening effects in a spin ensemble system (T2*) and keep the coherence for a relatively long time within T2 and/or T1 limitations of the qudits. While this refocusing scheme works well with the proof-of concept level for presented this qutrit system, there should be a discussion on how this number of refocusing pulses scales with the qudit dimension (d) and number of qudits (n). It would also be useful to note together the single pulse (or gate) fidelities required to achieve reasonable QFT process fidelities.**

We thank the Reviewer for these questions, giving us the opportunity to give further insight and perspective into the refocusing strategy. First, the Reviewer asks about the scaling of the refocusing with the qudit dimension *d*. This question was also posed by Reviewer 2 (question 4), and we reproduce here our answer:

> The refocusing block can be generalized for $d > 3$, with the depth of the block (time needed to implement it) scaling with $2d$ (for linear connectivity) or d (for loop or all-to-all connectivity). We updated the *Discussion* in the main text to address this:
>
> *"[…] with the time required for a refocusing block scaling as $2d\tau$ (where τ is the delay between pulses). […] For example, refocusing a system with d even and loop connectivity—linear connectivity with an additional connection between the edges— requires half the time than a system with linear connectivity."*

The Reviewer asks then also about the scaling of the refocusing with the number of qudits (*n*). This of course would require a scalable architecture with single qudits, as in the previous question. Assuming fast two-qudit gates, which could be possible in proposals based on coupling MQS through photons in circuit-QED architerctures, it is enough to refocus each qudit independently while they are uncoupled even if they are in an entangled state. Thus, the time needed to implement the refocusing strategy does not scale with the number of qudits if the qudits are inequivalent or in different coupled

resonators. We reproduce below the new parts of the *Discussion* section that deal with this specific question:

"[…] implementing two-qudit gates—essential for universal computation—necessitates the development of efficient interaction schemes such as resonant photon-exchange mediated coupling, which leverages tunable resonator frequencies to implement fast two-body operations between molecular units[33]. Refocusing protocols like the one introduced in this work could be integrated into these proposed schemes with fast multi-qudit gates, requiring only the refocusing of individual qudits during the idle (i.e. non-coupled) state of the multi-qudit register to implement complex algorithms like the extension of the QFT to multiple qudits."

There is one last question by the Reviewer about the single-pulse fidelities needed required to achieve a reasonable QFT. As we commented in our answer to question 4 by Reviewer 2, the number of refocusing blocks needed to perform a refocused implementation of an algorithm depends on the choice of decomposition of U_d in planar rotations and phase gates. For a specific decomposition choice, the figure in the next page shows a comparison of the estimated fidelities for the refocused implementation of the QFT for d=3,4,5,6 (the maximum number of states provided by the nuclear spin in ^{173}Yb(trensal)) as a function of the single-pulse fidelity (without inhomogeneous broadening). We introduced the error in the pulses as a dampening of the coherences, which seems to be the dominant error (for simplicity we assumed the same error on each pulse). Note that the single-pulse fidelity in this work (around 0.97, but it might be higher as this number includes also the effect of the tomography pulses) predicts refocused QFT fidelities (d=3) similar to those obtained experimentally. In this regard, fidelities of at least 0.92 for d=6 (using all nuclear spin states in one of the electronic spin manifolds of Yb(trensal)) would be in principle feasible in our setup even with no further single-pulse optimisation.

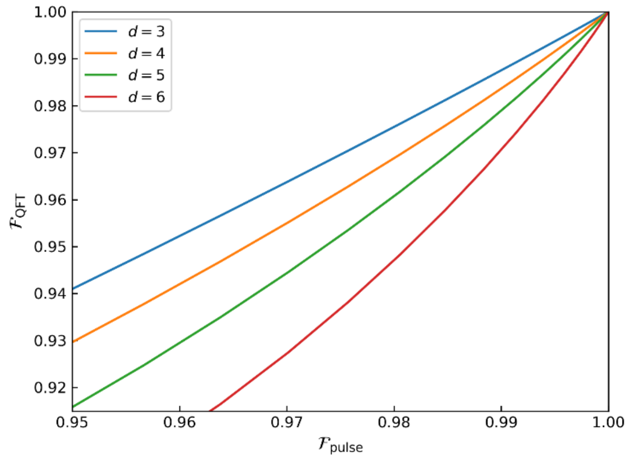

4. **In page 3, the statement "This demonstrates our coherent control of the qutrit state beyond Rabi experiments, with a gate fidelity F … = 0.97 ± 0.02." This sounds like the fidelity of state preparation and tomography, rather than the single-gate operation fidelity. I wonder whether the single gate fidelities are better than this value, and whether this can be enhanced with additional pulse sequences such as BB1 sequences (https://doi.org/10.1006/jmra.1994.1159).**

The Reviewer is right; the tomography is indeed part of the fidelity. Thus, we updated the text to clearly state it in the *Coherent control* section in *Results*:

"Our gate and sequence fidelity definitions always include the fidelity of the tomography procedure, which in fact is one of the limitations of our current setup."

We already tried implementing BB1 sequences for the preparation of initial states and we did not get a better preparation + tomography fidelity. This might suggest that the main limitation could be in the detection/tomography, which as the Reviewer pointed out also contributes to the fidelity. In this regard, we would like to explore the performance of a fully AWG-controlled platform in the future.

5. **Molecular spin qudits (MSQ) can be a promising platform for quantum information processing, and the authors implemented the QFT successfully, however, these results are confirmed using a spin ensemble. How can these results be further improved toward the level of single-spin manipulation and readout?**

We thank the Reviewer for this comment about future strategies towards molecular spin-based quantum processors. We are aware of single MQS manipulation and readout, including the implementation of Grover's algorithm, being demonstrated in a setup in which the MQS lies between two gold electrodes (reference 34). Thus, reaching the single-spin limit is possible. However, it is not clear how to scale up this setup to build a multiple MQS processor. For this reason, we focused in both the *Introduction* and the *Discussion* section in other strategies in which MQS are coupled through known scalable architectures like circuit-QED. As we replied to Reviewer 2 in question 1, in a very recent work available in arXiv (new reference 29) we were able to reach a single spin-photon coupling of 100 kHz in a circuit QED architecture. We consider also the possibility of controlling MQS through the electric field, and cite possible ways to address the challenge of placing a single MQS in the point of maximum coupling to photons in the superconducting circuit. We reproduce below the new parts in the *Discussion* section that deal with all these topics:

"A promising scalable strategy proposes the integration of MSQs into circuit-QED architectures by, for instance, embedding individual molecules within high-impedance superconducting resonators to achieve strong spin-photon coupling[29] and dispersive read-out. Alternative control mechanisms through electric fields are also under investigation, which potentially offer a more efficient and spatially-precise single spin-photon coupling[30,31]. Both approaches still pose the challenge of placing a single MSQ in specific points with maximum magnetic or electric coupling to photons in the superconducting circuit. This issue could be overcome via the growth of self-assembling monolayers or 2D molecular arrays on the circuit surface[52,53], or the directed assembly of nanostructures interfacing MSQs and circuits[54]."

In this text we added six new references, with the first three appearing first in the *Introduction* (answer to question 1 by Reviewer 2):

- [29] Rubín-Osanz, M. et al. Optimizing magnetic coupling in lumped element superconducting resonators for molecular spin qubits (2025).
  Link: https://doi.org/10.48550/arXiv.2511.00857

- [30] Liu, J. et al. Quantum coherent spin–electric control in a molecular nanomagnet at clock transitions. *Nat. Phys.* **17**, 1205–1209 (2021).
  Link: https://doi.org/10.1038/s41567-021-01355-4
- [31] Vaganov, M. V. et al. Chemical tuning of quantum spin–electric coupling in molecular magnets. *Nat. Chem.* **17**, 1903–1909 (2025).
  Link: https://doi.org/10.1038/s41557-025-01926-5
- [52] Tesi, L. et al. Modular approach to creating functionalized surface arrays of molecular qubits. *Adv. Mater.* **35**, 2208998 (2023).
  Link: https://doi.org/10.1002/adma.202208998
- [53] Urtizberea, A. et al. Vanadyl spin qubit 2D arrays and their integration on superconducting resonators. *Mater. Horiz.* **7**, 885–897 (2020).
  Link: https://doi.org/10.1039/C9MH01594A
- [54] Schreiber, R., Santiago, I., Ardavan, A. and Turberfield, A. J. Ordering gold nanoparticles with DNA origami nanoflowers. *ACS Nano* **10**, 7303–7306 (2016).
  Link: https://doi.org/10.1021/acsnano.6b03076

6. **In the Supplementary Information Chapter 2, the authors claim that inhomogeneous broadening mostly comes from a strain-induced distribution of the hyperfine coupling (A), rather than from a distribution of the Zeeman Splitting. I think record of the inhomogeneous broadening according to the external B0 field sweep will give more clear evidence. Is there corresponding data?**

We thank the Reviewer for his suggestion to check the origin of inhomogeneous broadening. Unfortunately, we don't have such B_0 field sweep data for the specific enrichment and magnetic dilution of the sample in this work, as we directly focused on implementing the QFT at B_0 = 0.2 T. However, we do have such data (B_0 = 0.1, 0.2 and 0.5 T) for a non-enriched sample (natural abundance of ^{173}Yb ≈ 16%) with a different magnetic dilution (1%) that we are studying as part of a different work. In this sample we observed minimal changes in the shape of the echo due to the field. Our simulations with only a distribution in the Zeeman coupling predict a stronger dependence with field. Conversely, simulations with only a distribution in the hyperfine couplings predict small changes among these B_0 values, consistent with the experiment.

However, we still consider that hyperfine alone is not enough to fit convincingly the results, implying that at least in this case there are other smaller contributions (including the Zeeman coupling) to inhomogeneous broadening. With this new information, we prefer to make a weaker claim in the *Discussion* section on the source of the hyperfine coupling by saying that the results in this work are <u>compatible</u> with a simple model that includes only a distribution in the hyperfine couplings, rather than including conclusions drawn from a different sample:

"We found the observed T_2^* in the two addressable transitions in this work to be compatible with a strain in the hyperfine couplings being the dominant mechanism (see SI)."

Reviewer #1 (Remarks to the Author):

The authors have provided exhaustive answers to all my questions and addressed the issues I raised. I am satisfied with the current status of the manuscript and recommend its publication in Nature Communications without further modification.

We thank the Reviewer for recommending the publication of the new version of the manuscript in Nature Communications.

Reviewer #2 (Remarks to the Author):

The authors have successfully addressed all my concerns and I think that the new version of the manuscript has improved. Therefore, I recommend its publication in its current form.

We thank the Reviewer for recommending the publication of the new version of the manuscript in Nature Communications.

Reviewer #3 (Remarks to the Author):

The authors have satisfactorily addressed my concerns and comments. I therefore recommend the manuscript for publication in Nature Communications.

We thank the Reviewer for recommending the publication of the new version of the manuscript in Nature Communications.
